# An OBSL1-Cul7^Fbxw8^ Ubiquitin Ligase Signaling Mechanism Regulates Golgi Morphology and Dendrite Patterning

**DOI:** 10.1371/journal.pbio.1001060

**Published:** 2011-05-10

**Authors:** Nadia Litterman, Yoshiho Ikeuchi, Gilbert Gallardo, Brenda C. O'Connell, Mathew E. Sowa, Steven P. Gygi, J. Wade Harper, Azad Bonni

**Affiliations:** 1 Department of Pathology, Harvard Medical School, Boston, Massachusetts, United States of America; 2 Program in Neuroscience, Harvard Medical School, Boston, Massachusetts, United States of America; 3 Department of Cell Biology, Harvard Medical School, Boston, Massachusetts, United States of America; University of Basel, Switzerland

## Abstract

The elaboration of dendrites in neurons requires secretory trafficking through the Golgi apparatus, but the mechanisms that govern Golgi function in neuronal morphogenesis in the brain have remained largely unexplored. Here, we report that the E3 ubiquitin ligase Cul7^Fbxw8^ localizes to the Golgi complex in mammalian brain neurons. Inhibition of Cul7^Fbxw8^ by independent approaches including Fbxw8 knockdown reveals that Cul7^Fbxw8^ is selectively required for the growth and elaboration of dendrites but not axons in primary neurons and in the developing rat cerebellum in vivo. Inhibition of Cul7^Fbxw8^ also dramatically impairs the morphology of the Golgi complex, leading to deficient secretory trafficking in neurons. Using an immunoprecipitation/mass spectrometry screening approach, we also uncover the cytoskeletal adaptor protein OBSL1 as a critical regulator of Cul7^Fbxw8^ in Golgi morphogenesis and dendrite elaboration. OBSL1 forms a physical complex with the scaffold protein Cul7 and thereby localizes Cul7 at the Golgi apparatus. Accordingly, OBSL1 is required for the morphogenesis of the Golgi apparatus and the elaboration of dendrites. Finally, we identify the Golgi protein Grasp65 as a novel and physiologically relevant substrate of Cul7^Fbxw8^ in the control of Golgi and dendrite morphogenesis in neurons. Collectively, these findings define a novel OBSL1-regulated Cul7^Fbxw8^ ubiquitin signaling mechanism that orchestrates the morphogenesis of the Golgi apparatus and patterning of dendrites, with fundamental implications for our understanding of brain development.

## Author Summary

The growth and elaboration of dendrites is an essential step in the establishment of neuronal circuits in the brain. Because dendrites house the receptive components of neurotransmission and actively integrate synaptic inputs, variations in dendrite architecture have important consequences for information processing. The development of dendrites relies on secretory trafficking through the Golgi apparatus. In this study, we have identified an E3 ubiquitin ligase, Cul7^Fbxw8^, that localizes to the Golgi apparatus in neurons. E3 ubiquitin ligases regulate the abundance of target proteins by directing ubiquitin-dependent proteolysis of specific targets. We have found that Cul7^Fbxw8^ operates at the Golgi apparatus to control Golgi integrity and dendrite patterning. We have also identified the cytoskeletal adaptor protein OBSL1 as an important regulator of Cul7^Fbxw8^ function in neurons. OBSL1 promotes the function of Cul7^Fbxw8^ by localizing Cul7 at the Golgi apparatus. Finally, we have found that Cul7^Fbxw8^ induces the ubiquitination and degradation of the Golgi protein Grasp65 to control Golgi morphology and dendrite elaboration. We conclude that the signaling cascade from OBSL1 to Cul7^Fbxw8^ to Grasp65 is an important means of regulating Golgi morphology and consequently the shape and size of dendrite arbors in neurons.

## Introduction

Establishing the uniquely complex and polarized morphology of neurons is essential for proper circuit development in the brain. The growth and elaboration of dendrite arbors determines access to synaptic partners and thus patterns neuronal connectivity. Secretory trafficking through the Golgi apparatus is selectively required for the elaboration of dendrites but not axon growth [1],[2]. Accordingly, manipulation of Golgi function triggers dramatic changes in dendrite growth and branching [1],[2]. However, the mechanisms that govern the morphology and function of the neuronal Golgi apparatus in the control of dendrite architecture have remained largely unexplored.

To regulate the development of distinct cellular compartments, including dendrites, axons, and synapses, neurons employ E3 ubiquitin ligases to regulate the abundance of proteins [3]–[8]. In mammalian brain neurons, the ubiquitin ligases Cdh1–anaphase promoting complex (Cdh1-APC) and Cdc20-APC operate in different cellular locales to control distinct aspects of neuronal morphogenesis [9]. Cdh1-APC acts in the nucleus, targeting the transcriptional regulators SnoN and Id2 for degradation, to limit axon growth [10]–[12]. In contrast, Cdc20-APC employs the centrosome as a signaling platform to promote dendrite elaboration [13]. These observations raise the intriguing possibility that yet to be identified mechanisms of spatially restricted ubiquitination operate at other major neuronal organelles and thereby control neuronal development.

Members of the large family of F-box proteins act as substrate specificity factors for the Skp1/Cul1/F-box (SCF) subfamily of cullin RING-type E3 ubiquitin ligases [14]–[16]. A number of F-box proteins have been implicated in neuronal survival and differentiation as well as synaptic transmission in the invertebrate and mammalian nervous systems [4],[5],[17]–[19]. However, the role of F-box proteins in neuronal morphogenesis and connectivity in the mammalian brain remains largely to be elucidated. F-box proteins are thought to provide spatial precision of substrate degradation in cells [20], suggesting that elucidation of F-box protein functions in neurons should uncover novel mechanisms of spatially restricted ubiquitin signaling in the establishment of neuronal circuitry.

Among F-box proteins, Fbxw8 is unique in that it primarily assembles with the scaffold protein Cul7 [21],[22]. Conversely, unlike the scaffold protein Cul1, which is thought to associate with all F-box proteins, Cul7 associates only with Fbxw8 [23],[24]. Thus, the Cul7^Fbxw8^ complex represents a unique yet poorly understood member of the cullin RING ligase family.

In this study, we identify Cul7^Fbxw8^ as an E3 ubiquitin ligase that localizes to the Golgi apparatus in neurons and is selectively required for growth and elaboration of dendrites. Strikingly, inhibition of Cul7^Fbxw8^ leads to impaired Golgi morphology and deficient secretory trafficking in neurons. Using an immunoprecipitation/mass spectrometry (IP/mass spec) screening approach, we identify the protein OBSL1 as a novel regulator of the ubiquitin ligase Cul7^Fbxw8^ in neurons. OBSL1 forms a physical complex with the scaffold protein Cul7, and thereby localizes Cul7 at the Golgi apparatus in neurons and promotes Golgi and dendrite morphogenesis. We also uncover the Golgi protein Grasp65 as a novel Cul7^Fbxw8^ substrate. Cul7^Fbxw8^ induces the ubiquitination and degradation of Grasp65, and thereby organizes the structure of the Golgi complex and drives dendrite elaboration. Together, our findings define a novel OBSL1-regulated Cul7^Fbxw8^ signaling pathway that orchestrates the morphogenesis of the Golgi apparatus and promotes dendrite elaboration and arborization.

## Results

### The E3 Ubiquitin Ligase Cul7^Fbxw8^ Drives Golgi Morphogenesis and Dendrite Patterning

We expressed Fbxw proteins tagged with green fluorescent protein (GFP) in primary granule neurons isolated from the rat cerebellar cortex. Granule neurons offer an ideal model system for studies of neuronal morphogenesis and connectivity in the mammalian brain [25],[26]. Following exit from the cell cycle, granule neurons migrate to the internal granule layer (IGL) in the developing cerebellar cortex, where they elaborate dendrites that grow and branch to establish robust dendrite arbors. Among the Fbxw proteins, we found that GFP-Fbxw8 had a unique localization, limited to a bean-shaped perinuclear area (Figure S1A). Upon co-expression of the Golgi marker mCherry-GT, which is targeted to the Golgi by a short N-terminal fragment of β1,4-galactosyltransferase [27], we found that GFP-Fbxw8 co-localized with mCherry-GT in granule neurons (Figure S1B). We therefore asked whether Fbxw8 might function at the Golgi apparatus in mammalian brain neurons.

**Figure 1:**
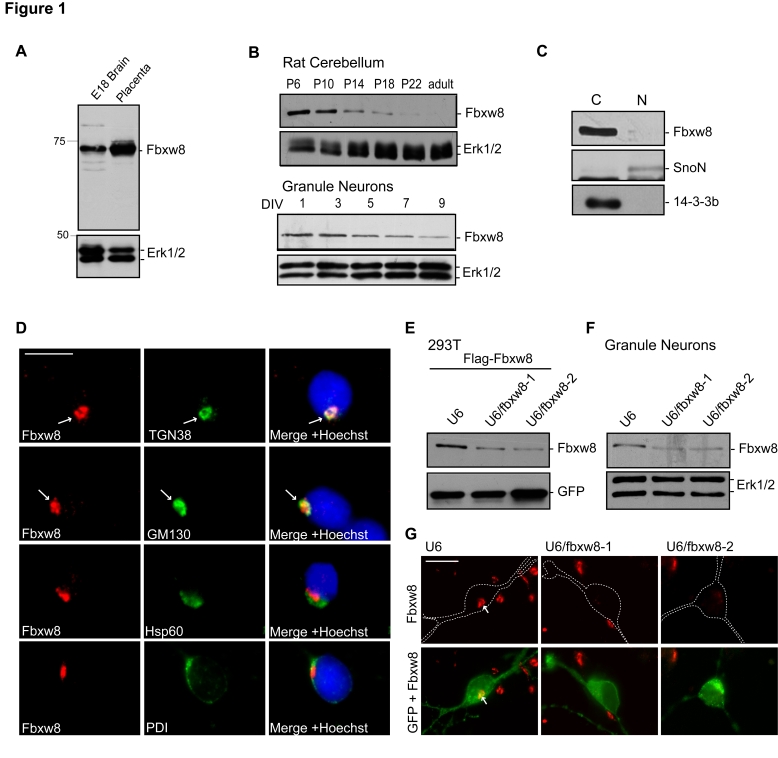
Fbxw8 is localized at the Golgi apparatus in neurons. (A) Lysates of embryonic brain and placenta were immunoblotted with the Fbxw8 and Erk1/2 antibodies. (B) Lysates of cerebellum from rat pups from P6 to adult and of primary P6 granule neurons cultured DIV1 to DIV9 were immunoblotted with the Fbxw8 and Erk1/2 antibodies. (C) Lysates of granule neurons were subjected to subcellular fractionation and immunoblotted with the Fbxw8, 14-3-3β, and SnoN antibodies, the latter two to mark cytoplasmic (C) and nuclear (N) fractions, respectively. (D) Granule neurons were subjected to immunocytochemistry with the Fbxw8 antibody together with the TGN38, GM130, Hsp60, or PDI antibody. DNA dye bisbenzimide (Hoechst 33258) was used to stain the nucleus. Arrows indicate localization of Fbxw8 at the Golgi apparatus. Scale bar = 10 µm. (E) Lysates of 293T cells transfected with the expression plasmids encoding Flag-Fbxw8 and GFP together with the U6/fbxw8-1, U6/fbxw8-2, or control U6 RNAi plasmid were immunoblotted with the Flag and GFP antibodies. (F) Lysates of granule neurons transfected with the U6/fbxw8-1, U6/fbxw8-2, or control U6 RNAi plasmid were immunoblotted with the Fbxw8 and Erk1/2 antibodies. (G) Granule neurons transfected at DIV2 with the U6/fbxw8-1, U6/fbxw8-2, or control U6 RNAi plasmid together with an expression plasmid encoding farnesylated GFP to label membranes were fixed at DIV5 and were subjected to immunocytochemistry using the GFP and Fbxw8 antibodies. Dotted lines represent tracing of transfected cells. Arrows indicate Golgi-localized Fbxw8 in transfected neurons. Scale bar = 10 µm. Fbxw8 knockdown reduced almost completely Fbxw8 immunofluorescence in neurons.

Among F-box proteins, Fbxw8 uniquely assembles with the scaffold protein Cul7 [21],[22], and unlike Cul1, which may associate with all F-box proteins, Cul7 associates only with Fbxw8 [23],[24]. Cul7^Fbxw8^ is highly expressed in placenta [21]–[23]. We found that Fbxw8 was also highly expressed in the developing rat brain (Figure 1A). In the rat cerebellum, Fbxw8 was abundantly expressed in the first two postnatal weeks, and its levels decreased thereafter (Figure 1B). Consistent with these results, Fbxw8 was highly expressed in primary granule neurons isolated from postnatal day 6 (P6) rat pups and cultured for 1 to 9 d in vitro, with expression decreasing over time with neuron maturation (Figure 1B). These results reveal that Fbxw8 is expressed in neurons with a temporal profile that coincides with the period of dendrite development.

We next characterized the subcellular localization of endogenous Fbxw8 in neurons. In immunoblotting analyses of fractionated lysates of granule neurons, Fbxw8 was enriched in the cytoplasmic fraction, which includes Golgi membranes (Figure 1C). In immunocytochemical analyses, we found that Fbxw8 immunofluorescence was perinuclear and bean-shaped in granule neurons, overlapping with the proteins GM130 and TGN38, which label cis- and trans-Golgi, respectively (Figure 1D). Fbxw8 did not co-localize with the mitochondrial protein Hsp60 or the endoplasmic reticulum (ER) protein PDI (Figure 1D). In granule neurons, the Golgi apparatus is often confined to the soma but can also extend into the proximal portion of a dendrite. Fbxw8 co-localized with Golgi markers in both cases. In other experiments, Fbxw8 also overlapped with the Golgi proteins GM130 and TGN38 in the soma and dendrites of hippocampal neurons, recapitulating the intricate morphology of this organelle in these neurons (Figure S1C).

To determine the specificity of Fbxw8 immunoreactivity in neurons, we used a plasmid-based method of RNA interference (RNAi) to acutely induce the knockdown of Fbxw8 [28]. Expression of short hairpin RNAs targeting two distinct regions of Fbxw8 (U6/fbxw8-1 or U6/fbxw8-2) efficiently reduced the expression of exogenous Fbxw8 in 293T cells and endogenous Fbxw8 in granule neurons (Figures 1E, 1F, S1D, and S1E). In immunocytochemical analyses, endogenous Fbxw8 immunofluorescence signal at the Golgi complex was almost completely abolished in granule neurons upon induction of Fbxw8 RNAi (Figure 1G). These results establish the specificity of the Fbxw8 immunoreactivity, and indicate that endogenous Fbxw8 is localized at the Golgi apparatus in neurons.

**Figure 2:**
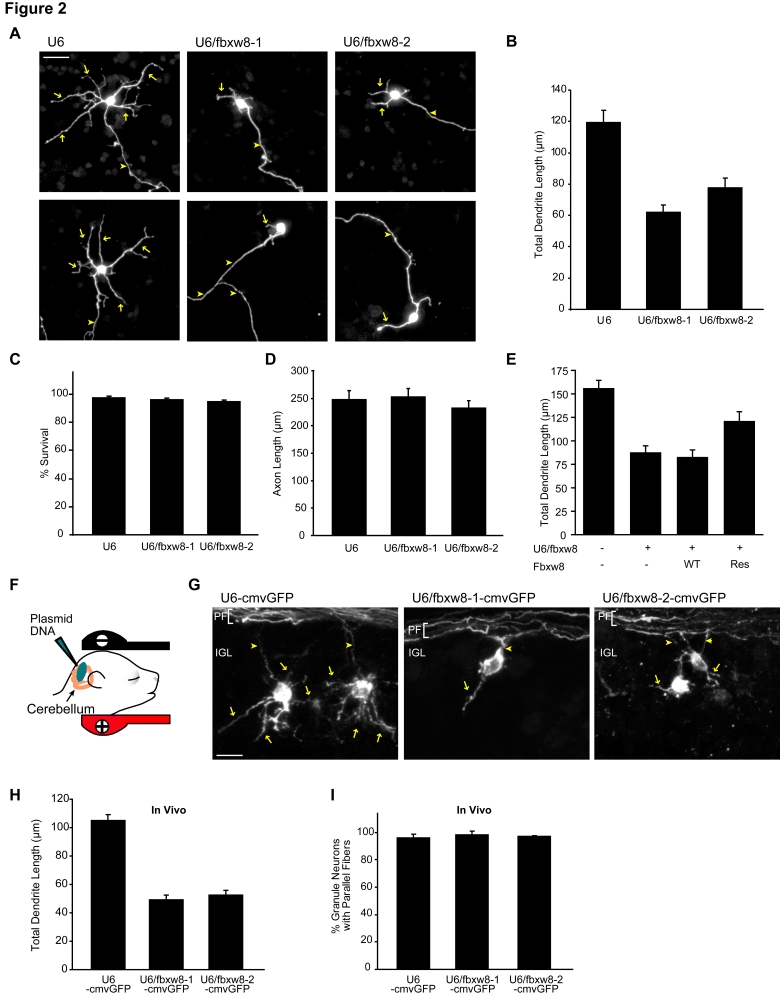
Fbxw8 promotes the elaboration of dendrites in primary mammalian neurons and in the cerebellar cortex in vivo. (A) Granule neurons transfected at DIV2 with the U6/fbxw8-1, U6/fbxw8-2, or control U6 RNAi plasmid together with the GFP expression plasmid were fixed at DIV5 and were subjected to immunocytochemistry with the GFP antibody. Representative images are shown. Arrows indicate dendrites and arrowheads indicate axons. Scale bar = 25 µm. Fbxw8 knockdown profoundly simplified dendrite arbors in granule neurons. (B) Quantification of total dendrite length in granule neurons analyzed as in (A) presented as mean+standard error of the mean. Total dendrite length was significantly reduced in Fbxw8 knockdown neurons as compared to control U6-transfected neurons (*p<*0.001, ANOVA followed by Bonferroni post hoc test; total neurons measured = 233). (C) Granule neurons transfected at DIV2 with the U6/fbxw8-1, U6/fbxw8-2, or control U6 plasmid together with the GFP expression plasmid were fixed at DIV5 and were subjected to immunocytochemistry with the GFP antibody and Hoechst to label the nuclei. Fbxw8 knockdown had little or no effect on cell survival in granule neurons (*n = *3). (D) Granule neurons were transfected 8 h after plating with the U6/fbxw8-1, U6/fbxw8-2, or control U6 plasmid together with the GFP expression plasmid, were fixed at DIV3, and were subjected to immunocytochemistry with the GFP antibody. Quantification of total axon length revealed that Fbxw8 knockdown had little or no effect on axon length (total neurons measured = 351). (E) Granule neurons transfected at DIV2 with the U6/fbxw8 or control U6 plasmid together with the expression plasmid encoding Fbxw8-WT, Fbxw8-Res, or control and the GFP expression plasmid were fixed at DIV8 and analyzed as in (A). Quantification of total dendrite length revealed that Fbxw8-Res but not Fbxw8-WT significantly increased dendrite length in the background of Fbxw8 RNAi (*p<*0.01, ANOVA followed by Bonferroni post hoc test; total neurons measured = 431). (F) Schematic of in vivo electroporation procedure. The U6/fbxw8-1-cmvGFP or U6/fbxw8-2-cmvGFP RNAi plasmid or control U6-cmvGFP plasmid was injected into the cerebellum in P3 rat pups, which were then subjected to electroporation. Animals were sacrificed 5 d later. (G) Cerebellar sections from P8 rat pups that were electroporated at P3 were subjected to immunohistochemistry with the GFP antibody. Representative images of transfected IGL granule neurons are shown for each condition. Granule neuron parallel fiber axons (PF) are shown oriented horizontally, ascending axons are marked by an arrowhead, and granule neuron dendrites are denoted by arrows. Scale bar = 10 µm. (H) Quantification of total dendrite length of IGL granule neurons analyzed as in (G). Total dendrite length was significantly reduced in Fbxw8 knockdown animals as compared to control animals (*p<*0.001, ANOVA followed by Bonferroni post hoc test; total neurons measured = 295). (I) Transfected granule neurons were counted in the IGL, and parallel fiber axons were counted in the molecular layer, as described previously [11]. No significant differences in parallel fiber number were observed in Fbxw8 knockdown animals as compared to control animals (total neurons measured = 567). Error bars indicate standard error of the mean.

In view of a reported requirement for secretory trafficking in dendrite development [1],[2], the intriguing localization of Fbxw8 at the Golgi complex led us to investigate its potential role in dendrite morphogenesis. We transfected granule neurons with the Fbxw8 RNAi or control U6 plasmid together with a GFP expression plasmid, and analyzed transfected neurons 3 d after transfection at day in vitro 5 (DIV5). Strikingly, we found that knockdown of Fbxw8 by the two distinct short hairpin RNAs profoundly impaired the growth and elaboration of dendrite arbors in granule neurons, leading to substantial reduction in the number of primary dendrites, the number of secondary and tertiary dendrite branches, and total dendrite length as compared to neurons transfected with the control U6 RNAi plasmid (Figures 2A, 2B, S2A, and S2B). Analyses of Fbxw8 knockdown neurons at later time points, at DIV8 and DIV10, revealed that simplification of dendrite arbors and reduction of total dendrite length were sustained (Figure S2C). Fbxw8 knockdown had little or no effect on cell survival (Figure 2C), suggesting that the impairment in dendrite elaboration was not secondary to dendrite degeneration or impaired health of neurons. In addition, axon growth was not hindered in Fbxw8 knockdown neurons as compared to neurons transfected with the control U6 RNAi plasmid (Figures 2D and S2D). Together, these results suggest a specific function for Fbxw8 in the elaboration and growth of dendrites in granule neurons.

To determine the specificity of the Fbxw8 RNAi-induced dendrite phenotype, we performed a rescue experiment. We generated an expression plasmid encoding a rescue form of Fbxw8 by introducing silent mutations in the cDNA encoding Fbxw8 protein designed to render it resistant to Fbxw8 RNAi (Fbxw8-Res). We confirmed that the expression of Fbxw8 short hairpin RNAs induced knockdown of Fbxw8 encoded by wild-type cDNA (Fbxw8-WT) but not Fbxw8-Res in cells (Figure S2E). In morphology assays, expression of Fbxw8-Res reversed the Fbxw8 RNAi-induced dendrite phenotype, restoring dendrite length to 77.5% of control-transfected neurons (Figures 2E and S2F). These results suggest that the Fbxw8 RNAi-induced dendrite phenotype is the result of specific knockdown of Fbxw8 rather than off-target effects of RNAi. In other experiments, we asked whether the expression of exogenous Fbxw8 in neurons might lead to a gain-of-function effect on dendrite growth. We found that expression of Fbxw8 in granule neurons at later developmental time points, when endogenous Fbxw8 levels are low, significantly increased dendrite length (Figure S3A and S3B). Collectively, based on loss-of-function and gain-of-function analyses, we conclude that Fbxw8 plays a critical role in dendrite elaboration.

We also characterized the role of Fbxw8 in dendrite morphogenesis in cerebral cortical and hippocampal neurons. Just as in granule neurons, Fbxw8 knockdown in both populations of neurons simplified their dendrite arbors, leading to a substantial reduction in dendrite length (Figure S3C and S3D). These results suggest that Fbxw8's function in driving the elaboration and growth of dendrites is generalized in mammalian brain neurons.

To determine the role of Fbxw8 in the context of the developing brain in the organism, we used an electroporation method to induce Fbxw8 knockdown in the cerebellum in vivo [10]. We injected a U6/fbxw8-1, U6/fbxw8-2, or U6 control RNAi plasmid that also encodes GFP into the cerebellum of P3 rat pups (Figure 2F). After electroporation, animals were allowed to develop until P8, and their cerebella were subjected to immunohistochemistry. Granule neurons in the IGL in the cerebellar cortex were identified by their distinctive “T-shaped” axons that form the parallel fibers and by their characteristically small soma size (Figure 2G). In control animals, IGL granule neurons had robust dendrite arbors (Figure 2G and 2H). In contrast, IGL granule neurons in Fbxw8 knockdown animals had simplified dendrite arbors (Figure 2G and 2H). Morphometric analyses revealed a significant reduction in IGL granule neuron primary dendrite number, dendrite branch number, and total dendrite length in Fbxw8 knockdown animals as compared to control animals (Figures 2H, S3E, and S3F). Importantly, Fbxw8 knockdown had little or no effect on the morphology or number of granule neuron parallel fiber axons in vivo (Figure 2I). Thus, our results suggest a physiological, cell-autonomous role for Fbxw8 in the elaboration of dendrites in the mammalian brain in vivo.

**Figure 3:**
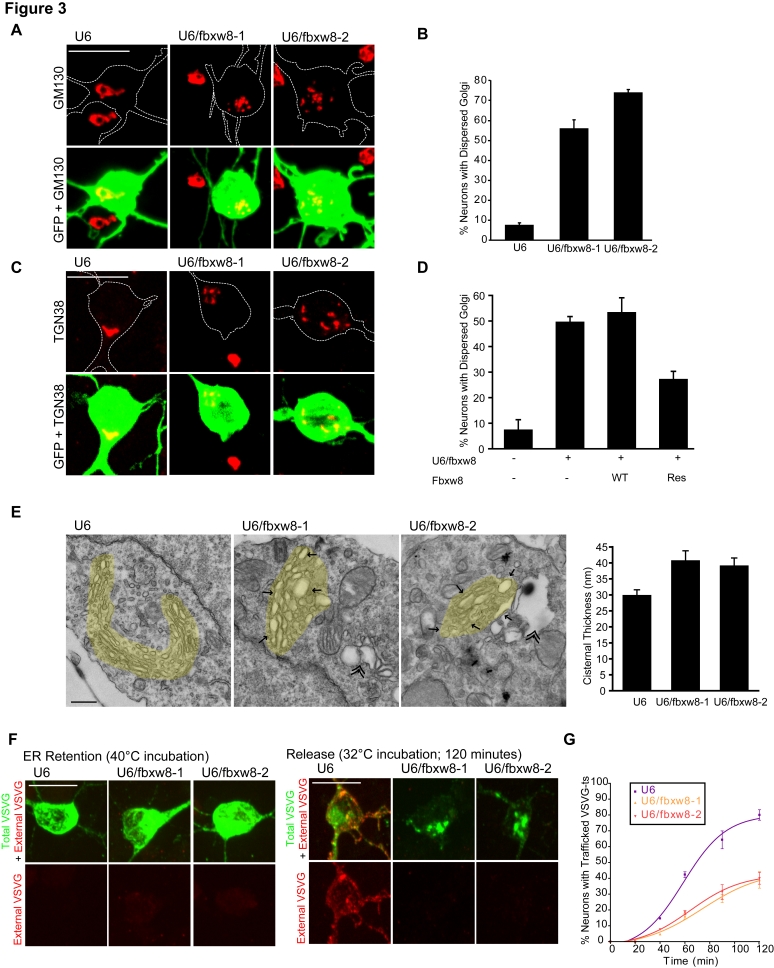
Fbxw8 is essential for morphogenesis of the Golgi apparatus in neurons. (A) Granule neurons transfected at DIV2 with the U6/fbxw8-1, U6/fbxw8-2, or control U6 RNAi plasmid together with the GFP expression plasmid were fixed at DIV5 and were subjected to immunocytochemistry with the GFP and GM130 antibodies. Representative images are shown. Scale bar = 10 µm. (B) Quantification of percentage of neurons analyzed as in (A) with dispersed Golgi, defined as having more than two GM130 puncta. Fbxw8 knockdown significantly increased the percentage of granule neurons with dispersed Golgi (*p<*0.001, ANOVA followed by Bonferroni post hoc test; *n = *5). (C) Granule neurons transfected as in (A) were subjected to immunocytochemistry with the GFP and TGN38 antibodies. Representative images are shown. Scale bar = 10 µm. Fbxw8 knockdown triggered the dispersion of the Golgi apparatus in neurons. (D) Granule neurons transfected at DIV2 with the U6/fbxw8 or control U6 plasmid together with the expression plasmid encoding Fbxw8-WT, Fbxw8-Res, or control vector and the GFP expression plasmid were analyzed as in (A). Quantification of percentage of neurons with dispersed Golgi revealed that Fbxw8-Res but not Fbxw8-WT significantly reduced the percent of cells with dispersed Golgi in the background of Fbxw8 RNAi (*p<*0.01, ANOVA followed by Bonferroni post hoc test; *n = *3). (E) Granule neurons transfected by nucleofection method with the U6/fbxw8-1, U6/fbxw8-2, or control U6 RNAi plasmid were collected after 4 d and processed for EM. In control immunocytochemical analyses, we found dispersed Golgi in 62%, 67%, and 5% of granule neurons transfected with the U6/fbxw8-1, U6/fbxw8-2, and control U6 RNAi plasmid, respectively. A representative EM image is shown for each condition. Yellow highlighting demarcates the Golgi stacks. Arrows indicate Golgi distortions in Fbxw8 knockdown neurons, including swelling and vesiculation of cisternae. Double arrowheads indicate vacuole formation. Scale bar = 500 nm. Right: quantification of swelling of Golgi cisternae as reflected by measurement of cisternal thickness. Fbxw8 knockdown significantly increased cisternal thickness (*p<*0.05, ANOVA followed by Bonferroni post hoc test; total cisternae measured = 241). (F) Granule neurons were transfected at DIV2 with the U6/fbxw8-1, U6/fbxw8-2, or the control U6 RNAi plasmid together with an expression plasmid encoding VSVG-ts-GFP, and after 3 d were incubated at 40°C for 8 h to allow for ER accumulation. Neurons were either fixed (left) or were moved to the ER to plasma membrane transport permissive temperature of 32°C and then fixed at various time points, including 120 min (right), and subjected to immunocytochemistry using the antibody that recognizes an extracellular epitope of VSVG-ts prior to permeabilization (red), and the GFP antibody after permeabilization to visualize total VSVG-ts (green). Representative images are shown. Scale bar = 10 µm. (G) Time course quantification of percentage of neurons analyzed as in (F) with VSVG-ts trafficked to the plasma membrane, as determined by positive external VSVG-ts signal. Fbxw8 knockdown significantly decreased the percentage of granule neurons with trafficked VSVG-ts at the 60-, 90-, and 120-min time points. Best fit curves as determined using a sigmoidal variable slope method are displayed (*p<*0.001, two-way ANOVA followed by Bonferroni post hoc test; *n = *3). Notably, dendrite growth was not compromised in Fbxw8 knockdown neurons in which VSVG-ts trafficked to the plasma membrane as compared to control neurons (Figure S4E). Error bars indicate standard error of the mean.

The growth and elaboration of dendrites is thought to be especially reliant on secretory trafficking [1],[2]. Because Fbxw8 selectively promotes dendrite growth and arborization and is localized to the Golgi apparatus in neurons, we hypothesized that Fbxw8 might impact the structure and function of this organelle. To test this possibility, we monitored the effect of Fbxw8 knockdown on Golgi morphology in granule neurons. We observed a single continuous bean-shaped Golgi in 93% of control granule neurons, as visualized by GM130 or TGN38 immunofluorescence (Figure 3A–3C). Remarkably, Fbxw8 knockdown led to dramatic dispersion of the Golgi complex, appearing as multiple, discontinuous GM130 or TGN38 immunoreactive puncta in a single neuron (Figure 3A and 3C). Dispersed Golgi, defined as two or more GM130 immunoreactive puncta per soma, were found in up to 74% of Fbxw8 knockdown granule neurons (Figure 3B). Notably, we found that Fbxw8 knockdown neurons with dispersed Golgi had significantly reduced total dendrite length compared to Fbxw8 knockdown neurons with nondispersed Golgi, suggesting that Golgi dispersion correlates tightly with reduced dendrite length in these neurons (Figure S4A). Importantly, expression of Fbxw8-Res, but not Fbxw8-WT, reversed the Fbxw8 RNAi-induced dispersion of the Golgi complex, suggesting that the Fbxw8 RNAi-induced Golgi phenotype is the result of specific knockdown of Fbxw8 (Figure 3D). In other experiments, Fbxw8 knockdown also triggered the dispersion of the Golgi apparatus in primary cerebral cortical and hippocampal neurons (Figure S4B and S4C). These results suggest a critical role for Fbxw8 in organizing the normal morphology of the Golgi apparatus in mammalian brain neurons.

We next characterized the effect of Fbxw8 knockdown on granule neuron Golgi at the ultrastructural level using electron microscopy (EM). In control U6-transfected neurons, EM revealed characteristic features of the Golgi apparatus, including neatly stacked organelle cisternae in a single ribbon (Figure 3E). In contrast, Fbxw8 knockdown neurons contained Golgi stacks that had distorted structure, marked by several abnormalities including swelling and vesiculation of cisternae and abnormal vacuole formation (Figure 3E). These findings establish that Fbxw8 is essential for the normal morphogenesis of the Golgi apparatus in neurons.

To determine the role of Fbxw8 in Golgi function, we assessed ER to plasma membrane trafficking in granule neurons. We expressed in granule neurons a vesicular stomatitis virus G-temperature sensitive mutant protein (VSVG-ts) tagged with GFP, and incubated neurons at 40°C to allow for accumulation in the ER [29],[30]. Neurons were next transferred to the permissive temperature of 32°C, which allows the VSVG-ts to exit the ER. To visualize VSVG-ts reaching the plasma membrane, prior to its permeabilization, neurons were subjected to immunocytochemical analysis using an antibody that recognizes an extracellular epitope of VSVG. After permeabilization, neurons were additionally probed with the GFP antibody to visualize total VSVG-ts-GFP. Neurons were fixed at various time points after ER release. After 2 h at 32°C, VSVG-ts reached the plasma membrane in nearly 80% of control U6-transfected neurons (Figure 3F and 3G). By contrast, VSVG-ts failed to reach the plasma membrane in more than half of Fbxw8 knockdown neurons after 2 h at 32°C (Figure 3F and 3G), and instead accumulated in the Golgi apparatus (Figure S4D). These results suggest that Fbxw8 is essential for Golgi function in neurons. Collectively, our data suggest that Fbxw8 plays a critical role in Golgi and dendrite morphogenesis in neurons.

In mammalian neurons, although much of the Golgi is housed in the soma and proximal dendrites, a population of Golgi outposts reside in dendrites. Golgi outposts are visualized in mammalian neurons using VSVG-ts after a 20°C block, which causes VSVG and other secretory cargo to accumulate in the Golgi apparatus [1]. We detected discrete VSVG-defined Golgi outposts throughout dendrites in primary granule neurons (Figure S5A). Knockdown of Fbxw8 reduced the number of VSVG-defined Golgi outposts (Figure S5A and S5B). Whereas there were five VSVG-defined Golgi outposts per neuron in control granule neurons, the number of VSVG-defined Golgi outposts was less than three per neuron upon Fbxw8 knockdown. These results suggest that, just as in the case of somatic Golgi, Fbxw8 promotes the morphogenesis of Golgi outposts.

We also assessed the effect of Fbxw8 knockdown on dendritic and axonal trafficking, using the dendritic protein transferrin receptor (TfR) and the axonal/presynaptic protein synapsin. Fbxw8 knockdown reduced the abundance of dendritic TfR (Figure S5C and S5D), which instead accumulated in the Golgi apparatus (Figure S5E). By contrast, Fbxw8 knockdown had little or no effect on the abundance of synapsin along the axon (Figure S5F and S5G). Together, these results suggest that Fbxw8 is selectively required for trafficking of dendritic components.

**Figure 4:**
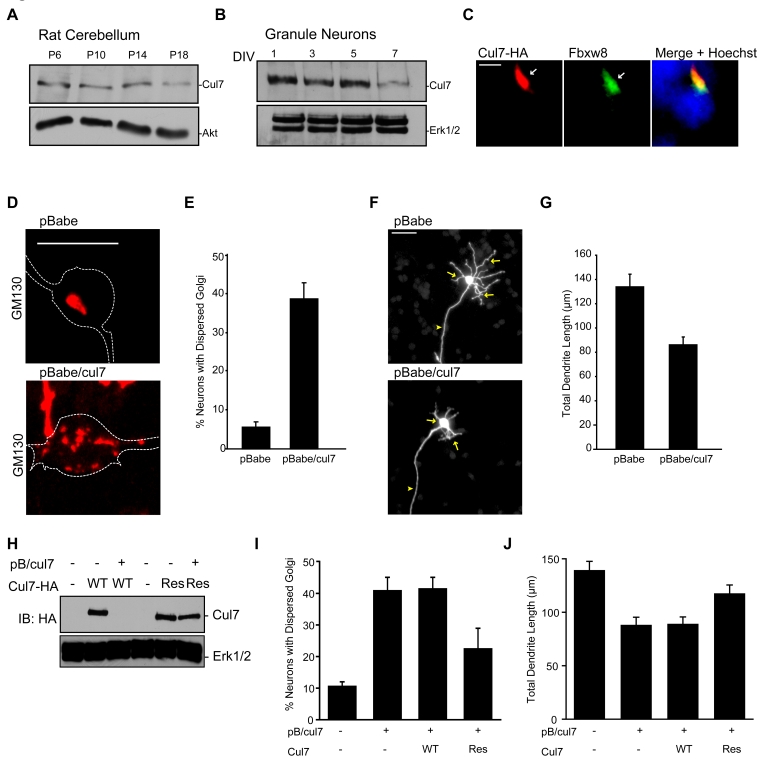
Cul7 is essential for morphogenesis of the Golgi apparatus and dendrites in neurons. (A) Lysates of cerebellum from rat pups from P6 to P18 were immunoblotted with Cul7 and Akt antibodies. (B) Primary P6 granule neurons cultured for DIV1 to DIV7 were immunoblotted with the Cul7 and Erk1/2 antibodies. (C) Granule neurons were transfected at DIV2 with the expression plasmid encoding Cul7-HA and subjected to immunocytochemistry with the HA and Fbxw8 antibodies. Arrows denote co-localization of Cul7 and Fbxw8. Scale bar = 2 µm. (D) Granule neurons transfected at DIV2 with the Cul7 RNAi or control pBabe plasmid together with the GFP expression plasmid analyzed as in Figure 3A. Representative images are shown. Scale bar = 10 µm. (E) Quantification of neurons analyzed as in (D) revealed that Cul7 knockdown significantly increased the number of granule neurons with dispersed Golgi (*p<*0.0005, *t* test; *n = *4). (F) Granule neurons transfected as in (D) were subjected to immunocytochemistry with the GFP antibody and analyzed as in Figure 2A. Representative images are shown. Scale bar = 25 µm. (G) Quantification of neurons analyzed as in (F) revealed that Cul7 knockdown significantly reduced total dendrite length in granule neurons (*p<*0.0001, *t* test; total neurons measured = 138). (H) Lysates of 293T cells transfected with the Cul7-WT-HA, Cul7-Res-HA, or control expression plasmid along with the Cul7 RNAi or control pBabe plasmid were immunoblotted with the indicated antibodies. (I) Granule neurons transfected at DIV2 with the pBabe/cul7 or control pBabe plasmid together with the expression plasmid encoding Cul7-WT, Cul7-Res, or control vector were analyzed as in (D). Quantification of percentage of neurons with dispersed Golgi revealed that Cul7-Res but not Cul7-WT significantly reduced the percent of cells with dispersed Golgi in the background of Cul7 RNAi (*p<*0.05, ANOVA followed by Bonferroni post hoc test; *n = *5). (J) Granule neurons transfected as in (I) were analyzed as in (F). Quantification of total dendrite length revealed that Cul7-Res but not Cul7-WT significantly increased dendrite length in the background of Cul7 RNAi (*p<*0.05, ANOVA followed by Bonferroni post hoc test; total neurons measured = 423). Error bars indicate standard error of the mean.

To characterize the mechanism by which Fbxw8 promotes neuronal Golgi and dendrite morphogenesis, we asked whether Fbxw8 operates in concert with the ubiquitin ligase scaffold protein Cul7 in neurons [21],[23]. Whereas other members of the cullin family of proteins act as scaffolds for numerous ubiquitin ligase substrate specificity factors, Cul7 is thought to associate exclusively with Fbxw8 among the F-box family of substrate specificity factors [24]. Cul7 was highly expressed in the developing rat brain and in granule neurons, with levels decreasing with neuronal maturation (Figure 4A and 4B). Thus, Cul7 is expressed in neurons with a temporal profile that coincides with Fbxw8 expression. Importantly, in analyses of the subcellular localization of Cul7, we found that Cul7 was co-localized with Fbxw8 at the Golgi complex in more than 80% of granule neurons (Figure 4C). Knockdown of Cul7 led to Golgi dispersion in granule neurons, as visualized by GM130 and TGN38 (Figures 4D, 4E, and S6A–S6D). Fbxw8 remained localized to dispersed Golgi elements in Cul7 knockdown neurons (Figure S6D). Cul7 knockdown also triggered simplification of dendrite arbors in granule neurons, leading to shorter dendrites (Figure 4F and 4G). Importantly, expression of the RNAi-resistant form of Cul7 (Cul7-Res) reversed Cul7 RNAi-induced Golgi dispersion and loss of dendrites in neurons (Figures 4H–4J and S6E), suggesting that the Cul7 RNAi-induced phenotypes result from specific knockdown of Cul7. In other analyses, we found that Cul7 knockdown neurons with dispersed Golgi had significantly reduced total dendrite length compared to Cul7 knockdown neurons with nondispersed Golgi, suggesting that Golgi dispersion correlates tightly with reduced dendrite length in these neurons (Figure S6F).

In ultrastructural analysis, Cul7 knockdown neurons harbored distorted Golgi structure, with swelling and vesiculation of cisternae (Figure S7A). We also found that Cul7 knockdown in postnatal rat pups led to simplification of dendrite arbors, with reduced total dendrite length in IGL granule neurons in the cerebellar cortex in vivo (Figure S7B–S7E). Together, these results reveal that Cul7 knockdown phenocopies the effect of Fbxw8 knockdown on both Golgi morphology and dendrite elaboration. In gain-of-function analyses, expression of exogenous Cul7 in granule neurons at later developmental time points, when endogenous Cul7 levels are diminished, led to an increase in dendrite length (Figure S3A and S3B). Taken together, these findings suggest that Cul7 and Fbxw8 act in a common pathway to promote Golgi and dendrite morphogenesis.

We also determined the effect of expression of a dominant interfering form of Fbxw8, owing to deletion of the F-box domain (Fbxw8ΔFbox), on Golgi and dendrite morphogenesis in neurons. We found that expression of Fbxw8ΔFbox induced Golgi dispersion and reduced dendrite length in granule neurons (Figure S8A–S8D). These data both corroborate the results of Fbxw8 RNAi-induced Golgi and dendrite phenotypes and highlight the importance of the F-box domain in Fbxw8 function in neurons. Collectively, our results suggest that Fbxw8 and Cul7 act as components of the ubiquitin ligase Cul7^Fbxw8^ to promote Golgi and dendrite morphology.

### The Cytoskeletal Adaptor Protein OBSL1 Localizes the Scaffold Protein Cul7 at the Golgi Apparatus and Thereby Promotes Golgi and Dendrite Morphogenesis

**Figure 5:**
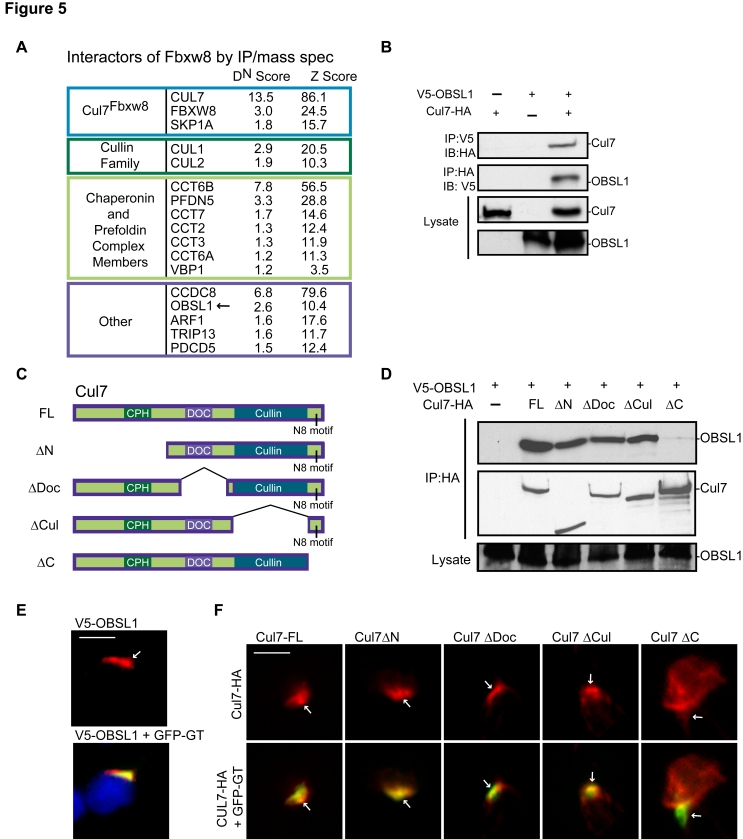
The cytoskeletal adaptor protein OBSL1 forms a physical complex with the scaffold protein Cul7. (A) Lysates of 293T cells harboring an inducible HA-Fbxw8 lentivirus were immunoprecipitated with the HA antibody and subjected to proteomic analysis using LC-MS/MS. CompPASS was utilized to interrogate datasets and assign the *D* ^N^ and *Z* scoring metrics. Proteins with a *D* ^N^ score greater than 1 and a *Z* score greater than 3.5 were considered HCIPs. We confirmed that endogenous Fbxw8 and endogenous Cul7 form a complex in cells (Figure S6G). (B) Lysates of 293T cells transfected with expression plasmids encoding V5-OBSL1 and Cul7-HA or the control vectors were immunoprecipitated (IP) with the V5 or HA antibodies. Immunoprecipitates and lysates were immunoblotted (IB) with the V5 and HA antibodies. (C) Domain map of full-length (FL) Cul7 protein and deletion mutant proteins. Cul7 consists of a large N-terminal domain unique among the cullin family that contains a CPH domain, a DOC domain, a cullin domain, and an extreme C-terminal region that contains a neddylation motif. (D) Lysates of 293T cells transfected with expression plasmids encoding V5-OBSL1 and full-length Cul7-HA, deletion mutants, or the control vector were immunoprecipitated with the HA antibody. Immunoprecipitates and lysates were immmunoblotted with the HA and V5 antibodies. (E) Granule neurons were transfected with the expression plasmids encoding V5-OBSL1 and the Golgi marker, GFP-GT, and subjected to immunocytochemistry with the V5 and GFP antibodies. Arrow denotes co-localization of OBSL1 and GFP-GT. Scale bar = 5 µm. (F) Granule neurons were transfected with the expression plasmids encoding Cul7-HA or deletion mutants and the Golgi marker, GFP-GT, and subjected to immunocytochemistry with the HA and GFP antibodies. Arrows denote the Golgi apparatus as labeled by GFP-GT. Scale bar = 5 µm.

Having identified an essential role for the ubiquitin ligase Cul7^Fbxw8^ ubiquitin signaling pathway in Golgi morphogenesis and dendrite patterning in neurons, we next addressed the major question of how the function of neuronal Cul7^Fbxw8^ is controlled. To identify novel regulators of Cul7^Fbxw8^ in an unbiased manner, we utilized an IP/mass spec screening approach [31]. We immunopurified Fbxw8 complexes from 293T cells that were infected with an inducible HA-Fbxw8 lentivirus. Purified Fbxw8 complexes were next subjected to liquid chromatography–tandem mass spectrometry (LC-MS/MS) analyses to identify Fbxw8-associated proteins. We interrogated datasets using the platform Comparative Proteomics Analysis Software Suite (CompPASS) to assign scoring metrics, *D* ^N^ and *Z* scores, for parallel mass spectral studies. The *D* ^N^ score is similar to the conventional *Z* score, but it also incorporates the frequency of the observed interactor, its abundance, and the reproducibility of the interaction [31]. Proteins with a *D* ^N^ score greater than 1 and a *Z* score greater than 3.5 were considered high-confidence candidate-interacting proteins (HCIP) (Figure 5A). Proteins reproducibly identified as HCIPs in our analyses included those established to interact with Fbxw8, including Cul7 and Skp1A, validating the IP/mass spec approach. We also identified five subunits of the CCT (chaperonin containing TCP1) complex in association with Fbxw8. The CCT complex has been implicated in the proper folding of WD40 domain proteins [32], suggesting that Fbxw8 may also employ CCT for folding.

Among novel interactors of Fbxw8, the cytoskeletal adaptor protein OBSL1 was of particular interest because of a recently identified genetic relationship between OBSL1 and Cul7. Both OBSL1 and the scaffold protein Cul7 are mutated in the rare inherited human genetic disorder 3M syndrome, characterized by growth retardation [33]–[35]. In view of the genetic link between OBSL1 and Cul7 in human disease, our IP/mass spec data raised the possibility that OBSL1 might represent a true interactor of Cul7^Fbxw8^. In reciprocal co-immunoprecipitation analyses, we found that OBSL1 formed a physical complex with Cul7 in cells (Figure 5B). Cul7 is composed of a large N-terminal region that contains a CPH (conserved within Cul7, Parc, Herc2) domain, a DOC domain, a cullin domain, and a neddylation motif in the extreme C-terminus (Figure 5C). In structure–function analyses, the extreme C-terminus of Cul7 was required for the interaction of Cul7 and OBSL1 (Figure 5D). In in vitro binding assays, a recombinant form of the C-terminal domain of Cul7 that includes the cullin domain (glutathione S-transferase [GST]–Cul7C) formed a complex with in vitro transcribed and translated OBSL1 (Figure S9A), suggesting that Cul7 and OBSL1 interact directly.

**Figure 6:**
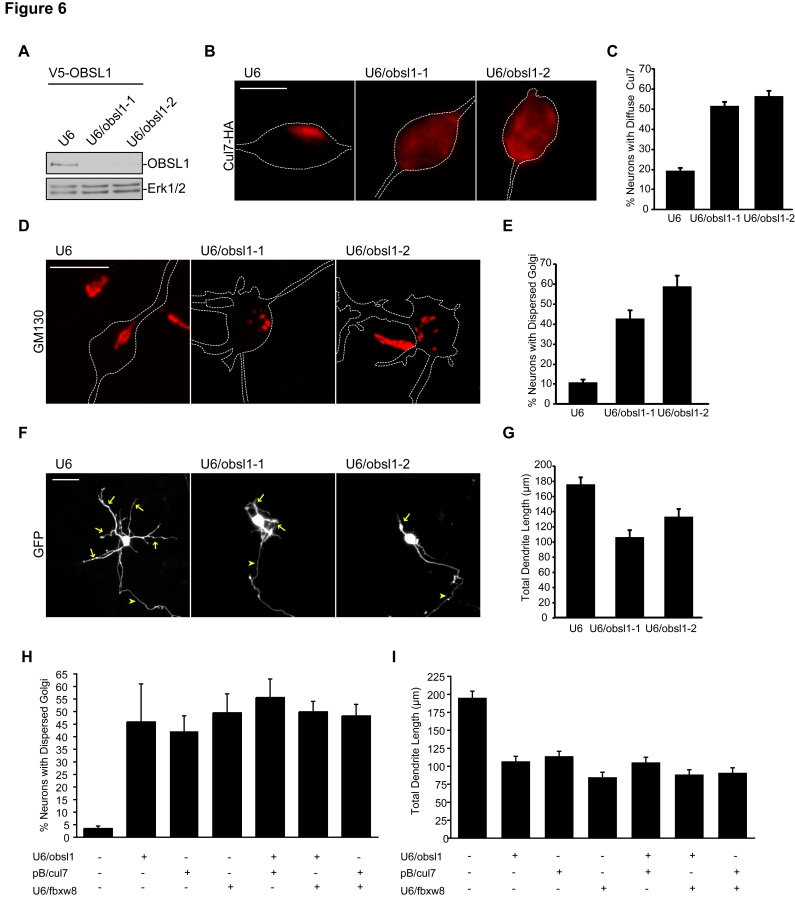
The cytoskeletal adaptor protein OBSL1 localizes the scaffold protein Cul7 to the Golgi apparatus and promotes Golgi morphology and dendrite elaboration. (A) Lysates of 293T cells transfected with an expression plasmid encoding V5-OBSL1 and U6/obsl1-1, U6/obsl1-2, or control U6 RNAi were immunoblotted with V5 and Erk1/2 antibodies. (B) Granule neurons transfected at DIV1 with U6/obsl-1, U6/obsl1-2, or control U6 RNAi plasmid together with the GFP and Cul7-HA expression plasmids were fixed at DIV6 and subjected to immunocytochemistry with the HA and GFP antibodies. Representative images are shown. Scale bar = 5 µm. (C) For quantification of Cul7 localization, we defined diffuse Cul7 staining as occurring when Cul7 was found distributed evenly throughout the soma and in the dendrites. Quantification of percentage of neurons analyzed as in (B) with diffuse Cul7 staining revealed that OBSL1 knockdown significantly increased the percentage of granule neurons with diffuse Cul7 staining (*p<*0.001, ANOVA followed by Bonferroni post hoc test; *n = *3). (D) Granule neurons transfected at DIV1 with U6/obsl-1, U6/obsl1-2, or control U6 RNAi plasmid together with the GFP expression plasmid were fixed at DIV6 and analyzed as in Figure 3A. Representative images are shown. Scale bar = 10 µm. (E) Quantification of neurons analyzed as in (D) revealed that OBSL1 knockdown significantly increased the percentage of granule neurons with dispersed Golgi (*p<*0.01, ANOVA followed by Bonferroni post hoc test; *n = *4). (F) Granule neurons transfected as in (D) were subjected to immunocytochemistry with the GFP antibody and analyzed as in Figure 2A. Arrows indicate dendrites; arrowheads indicate axons. Representative images are shown. Scale bar = 25 µm. (G) Quantification of neurons analyzed as in (F) revealed that OBSL1 knockdown significantly reduced total dendrite length in granule neurons (*p<*0.01, *t* test; total neurons measured = 272). (H) Granule neurons transfected with the OBSL1 RNAi, Cul7 RNAi, Fbxw8 RNAi or control U6 RNAi plasmid, separately or in combination as indicated, together with the GFP expression plasmid were analyzed as in (D). The percentage of cells with dispersed Golgi was not significantly different upon Fbxw8 knockdown, Cul7 knockdown, and OBSL1 knockdown separately or in combination (*n = *3). (I) Granule neurons transfected as in (H) and analyzed as in (F). Total dendrite length was not significantly different upon Fbxw8 knockdown, Cul7 knockdown, and OBSL1 knockdown separately or in combination (total neurons measured = 714). Error bars indicate standard error of the mean.

In view of the function of OBSL1 as a cytoskeletal adaptor [36], the interaction of OBSL1 with Cul7 led us to investigate the role of OBSL1 in the localization of Cul7 at the Golgi apparatus. OBSL1 was expressed in granule neurons, and its levels decreased with neuronal maturation (Figure S9B). OBSL1 was localized in the perinuclear region overlapping with the Golgi marker GFP-GT, suggesting that OBSL1 is present at the Golgi apparatus in neurons (Figure 5E). In structure–function analyses, although full-length Cul7 as well as deletion mutants lacking the N-terminus, the DOC domain, or the cullin domain were enriched at the Golgi apparatus, the Cul7 mutant protein lacking the extreme C-terminus was expressed diffusely throughout the cytoplasm and did not accumulate at the Golgi apparatus (Figure 5F). Thus, the extreme C-terminus of Cul7, which is required for the binding of Cul7 with OBSL1, also mediates Cul7 localization to the Golgi apparatus. Importantly, OBSL1 knockdown in neurons led to the diffuse localization of Cul7 throughout the cytoplasm, away from the Golgi apparatus (Figures 6A–6C, S9C, and S9D), suggesting that OBSL1 plays a critical role in the subcellular localization of Cul7. Cul7 was not mislocalized in all OBSL1 knockdown neurons, raising the possibility that OBSL1-independent mechanisms may also contribute to the control of Cul7 subcellular localization. In control experiments, OBSL1 knockdown did not significantly alter Cul7 protein levels in neurons (Figure S9E). Notably, Fbxw8 remained localized at the Golgi apparatus in OBSL1 knockdown neurons (Figure S9F), suggesting that OBSL1 specifically localizes Cul7 to the Golgi complex. In addition, in contrast to the mislocalization of Cul7 in OBSL1 knockdown neurons, Cul7 remained localized at the Golgi apparatus upon Fbxw8 knockdown or Fbxw8ΔFbox expression (Figure S10A and S10B). Likewise, OBSL1 remained localized at the Golgi apparatus in neurons upon inhibition of Cul7^Fbxw8^ (Figure S10C–S10E). Collectively, these results reveal that OBSL1 specifically localizes the scaffold protein Cul7 to the Golgi apparatus in neurons.

Having identified a function for OBSL1 in the localization of Cul7 at the Golgi apparatus, we next determined the role of OBSL1 in the control of Golgi morphology and dendrite development. Since Cul7^Fbxw8^ plays a critical role in Golgi morphology and dendrite elaboration, OBSL1 knockdown would be predicted to induce dispersion of the Golgi apparatus and impair dendrite morphogenesis owing to the mislocalization of Cul7 away from the Golgi upon OBSL1 knockdown. Consistent with this prediction, OBSL1 knockdown led to Golgi dispersion in granule neurons (Figure 6D and 6E). In ultrastructural EM analyses, OBSL1 knockdown neurons displayed distorted Golgi structure, with swelling and vesiculation of cisternae (Figure S11A). In neuronal morphology assays, we found that OBSL1 knockdown impaired the elaboration of dendrite arbors in granule neurons, leading to reduction of total dendrite length (Figure 6F and 6G). Importantly, in rescue experiments, expression of an RNAi-resistant form of OBSL1 (OBSL1-Res), but not OBSL1 encoded by wild-type cDNA (OBSL1-WT), reversed the OBSL1 RNAi-induced Golgi dispersion and dendrite loss in granule neurons (Figure S11B–S11E), suggesting that the OBSL1 RNAi-induced phenotypes result from specific knockdown of OBSL1. In other experiments, we found that OBSL1 knockdown in postnatal rat pups led to simplification of dendrite arbors, leading to reduced total dendrite length in IGL granule neurons in the cerebellar cortex in vivo (Figure S7B–S7E). Together, these results reveal that OBSL1 knockdown phenocopies the effects of inhibition of Cul7^Fbxw8^ on both Golgi morphology and dendrite elaboration.

To further clarify the functional relationship between OBSL1, Cul7, and Fbxw8 in dendrite morphogenesis and Golgi morphology, we analyzed the effect of knockdown of each of these molecules separately and in combination on Golgi and dendrite morphogenesis. We found that the effects of knockdown of OBSL1, Cul7, and Fbxw8 on Golgi and dendrite morphology were not additive (Figure 6H and 6I), suggesting that OBSL1, Cul7, and Fbxw8 act in a common pathway. Collectively, our results suggest that OBSL1 regulates the ubiquitin ligase Cul7^Fbxw8^ to promote Golgi and dendrite morphology.

### Identification of the Golgi Protein Grasp65 as a Novel Substrate of Cul7^Fbxw8^ in the Control of Golgi Morphogenesis and Dendrite Patterning

A major question that remained to be addressed is how Cul7^Fbxw8^ regulates Golgi and dendrite morphogenesis. We reasoned that as an E3 ubiquitin ligase, Cul7^Fbxw8^ might induce the ubiquitination and subsequent degradation of a substrate protein at the Golgi apparatus and thereby regulate Golgi and dendrite morphogenesis. Because Cul7^Fbxw8^ promotes secretory trafficking and dendrite elaboration, substrates of Cul7^Fbxw8^ would be predicted to restrict secretory trafficking and dendrite growth. The Golgi stacking protein Grasp65 restricts secretory trafficking in normal rat kidney cells [37], raising the possibility that Grasp65 might represent a candidate substrate of Cul7^Fbxw8^. We found that inhibition of Grasp65 by RNAi accelerates secretory trafficking via the Golgi apparatus in granule neurons (Figure S12A–S12D), suggesting that, just as in non-neuronal cells, Grasp65 restricts secretory trafficking in neurons.

**Figure 7:**
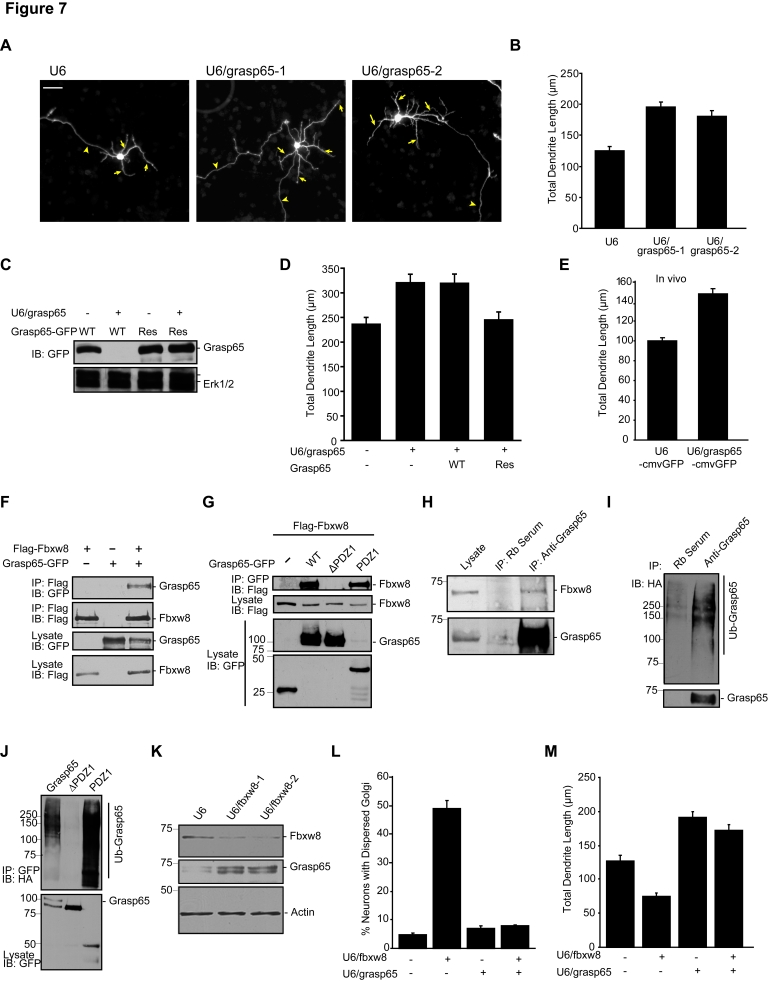
Cul7^Fbxw8^ triggers the ubiquitination and degradation of Grasp65 to promote Golgi morphogenesis and drive dendrite elaboration in neurons. (A) Granule neurons transfected with the U6/grasp65-1, U6/grasp65-2, or control U6 RNAi plasmid analyzed as in Figure 2A. Scale bar = 25 µm. (B) Quantification of neurons analyzed as in (A) revealed that total dendrite length was significantly increased in Grasp65 knockdown neurons as compared to control U6-transfected neurons (*p<*0.001, ANOVA followed by Bonferroni post hoc test; total neurons measured = 209). (C) Lysates of 293T cells transfected with the Grasp65-WT-GFP or Grasp65-Res-GFP expression plasmids along with the Grasp65 RNAi or control U6 plasmid were immunoblotted (IB) with the indicated antibodies. (D) Granule neurons transfected at DIV2 with the U6/grasp65 or control U6 plasmid together with the expression plasmid encoding Grasp65-WT, Grasp65-Res, or control vector were analyzed as in (A). Quantification of total dendrite length revealed that Grasp65-Res but not Grasp65-WT significantly decreased dendrite length in the background of Grasp65 RNAi (*p<*0.01, ANOVA followed by Bonferroni post hoc test; total neurons measured = 409). (E) Cerebella from P8 rat pups after in vivo electroporation of the U6/grasp65-cmvGFP or control U6-cmvGFP plasmid at P3 were analyzed as in Figure 2F. Total dendrite length in IGL granule neurons was significantly increased in Grasp65 knockdown animals as compared to control animals (*p<*0.0001, *t* test; total neurons measured = 168). (F) Lysates of 293T cells transfected with expression plasmids encoding Flag-Fbxw8 and Grasp65-GFP or the control vectors were immunoprecipitated (IP) with the Flag antibody. Immunoprecipitates and lysates were immunoblotted with the indicated antibodies. (G) Lysates of 293T cells transfected with the expression plasmids encoding Flag-Fbxw8 and Grasp65-GFP, mutants Grasp65ΔPDZ1 or PDZ1, or the control vector were immunoprecipitated with the GFP antibody. Immunoprecipitates and lysates were immunoblotted with the indicated antibodies. (H) Lysates of cortical neurons were immunoprecipitated with the Grasp65 antibody or pre-immune serum followed by immunoblotting with the Grasp65 and Fbxw8 antibodies. Endogenous Grasp65 co-immunoprecipitates with endogenous Fbxw8 in neurons. (I) Lysates of granule neurons transfected with an expression plasmid encoding HA–ubiquitin (Ub) were immunoprecipitated with the Grasp65 antibody or pre-immune serum followed by immunoblotting with the HA and Grasp65 antibodies. (J) Lysates of granule neurons transfected with expression plasmids encoding Grasp65-GFP or its mutants together with the HA-ubiquitin expression plasmid were immunoprecipitated with the GFP antibody. Lysates and immunoprecipitates were immunoblotted with the GFP and HA antibodies, respectively. The PDZ1 domain is necessary and sufficient for Grasp65 to undergo polyubiquitination in neurons. (K) Lysates of granule neurons that had been transfected by nucleofection method with the U6/fbxw8-1, U6/fbxw8-2, or control U6 RNAi plasmid were immunoblotted with the Fbxw8, Grasp65, and Actin antibodies. Knockdown of Fbxw8 led to increased levels of endogenous Grasp65 in neurons. (L) Granule neurons were transfected with the U6/fbxw8, U6/grasp65, or control U6 plasmid, separately or in combination, and analyzed as in Figure 3A. Grasp65 knockdown significantly suppressed Fbxw8 RNAi-induced Golgi dispersion (*p<*0.001, ANOVA followed by Bonferroni post hoc test; *n = *3). (M) Granule neurons transfected as in (L) and analyzed as in Figure 2A. Simultaneous knockdown of Grasp65 and Fbxw8 significantly increased dendrite length compared to control U6 or Fbxw8 knockdown alone (*p<*0.001, ANOVA followed by Bonferroni post hoc test; total neurons measured = 330). Error bars indicate standard error of the mean.

In neuronal morphology assays, we found that dendrites in Grasp65 knockdown granule neurons were longer and more highly branched than dendrites in control neurons (Figures 7A, 7B, S12E, and S12F). Importantly, expression of an RNAi-resistant rescue form of Grasp65 (Grasp65-Res), but not Grasp65 encoded by wild-type cDNA, reversed the Grasp65 RNAi-induced dendrite phenotype, suggesting that the Grasp65 RNAi-induced dendrite phenotype is the result of specific knockdown of Grasp65 (Figure 7C and 7D). We also found that Grasp65 knockdown in postnatal rat pups led to more elaborate dendrite arbors, with increased total dendrite length in IGL granule neurons in the cerebellar cortex in vivo (Figures 7E, S12G, and S12H). These results suggest that, consistent with the possibility that Grasp65 might represent a substrate of Cul7^Fbxw8^, Grasp65 inhibits the elaboration and arborization of dendrites in neurons.

We next performed biochemical analyses to determine whether Grasp65 might act as a substrate of Cul7^Fbxw8^. We first assessed the ability of Grasp65 to interact with Fbxw8. In complementary reciprocal co-immunoprecipitation analyses, Fbxw8 and Grasp65 formed a physical complex in cells (Figure 7F). Notably, the Golgi protein Grasp55, which is closely related to Grasp65 [38], failed to interact with Fbxw8 (Figure S13A), highlighting the specificity of the interaction between Grasp65 and Fbxw8. Grasp65 is composed of two tandem N-terminal PDZ-like domains (PDZ1, PDZ2) followed by a C-terminal phospho-serine-rich domain [39],[40]. We found that a Grasp65 mutant protein that lacked PDZ1 (Grasp65ΔPDZ1) failed to interact with Fbxw8 in cells (Figure 7G). Conversely, the PDZ1 domain on its own interacted with Fbxw8 (Figure 7G). Thus, the PDZ1 domain appears to be both necessary and sufficient for the interaction of Grasp65 with Fbxw8. Together, these data suggest that Fbxw8 and Grasp65 form a physical complex.

We next assessed the ability of Fbxw8 to catalyze the ubiquitination of Grasp65 in cells. Expression of Fbxw8 triggered the accumulation of polyubiquitinated Grasp65 in 293T cells (Figure S11B). Importantly, Fbxw8 failed to induce the ubiquitination of the Grasp65 mutant protein that is unable to interact with Fbxw8, Grasp65ΔPDZ1 (Figure S13B). In other experiments, Fbxw8 failed to induce the ubiquitination of the Grasp65-related protein Grasp55 (data not shown), correlating with ability of Fbxw8 to interact with Grasp65 but not Grasp55. We also found that expression of Fbxw8, but not Fbxw8ΔFbox, induced the downregulation of Grasp65 in cells (Figure S13C). However, Fbxw8 failed to induce the downregulation of Grasp65ΔPDZ1 (Figure S13C), suggesting that the PDZ1 domain in Grasp65 is important for Fbxw8-induced ubiquitination and degradation of Grasp65.

We next determined whether Grasp65 is a substrate of Cul7^Fbxw8^ in neurons. Consistent with the interpretation that Grasp65 might represent a substrate of Cul7^Fbxw8^, endogenous Fbxw8 interacted with endogenous Grasp65 in neurons (Figure 7H). We also found that endogenous Grasp65 undergoes polyubiquitination in granule neurons (Figure 7I). In other experiments, we detected ubiquitinated conjugates of full-length Grasp65 and the PDZ1 domain on its own but not Grasp65ΔPDZ1 in granule neurons (Figure 7J). Thus, just as in 293T cells, the PDZ1 domain in Grasp65, which binds to Fbxw8, facilitates the ubiquitination of Grasp65 in neurons. Importantly, knockdown of endogenous Fbxw8 led to the accumulation of endogenous Grasp65 protein in granule neurons (Figures 7K and S13D). Taken together, these results suggest that the ubiquitin ligase Cul7^Fbxw8^ triggers the ubiquitination and consequent degradation of Grasp65 in neurons.

To assess whether Grasp65 is a physiologically relevant substrate of Cul7^Fbxw8^ in the control of Golgi and dendrite morphology, we performed epistasis analysis. In analyses of Golgi morphology, Grasp65 knockdown prevented Fbxw8 knockdown-induced dispersion of the Golgi complex in granule neurons (Figure 7L). We also found that Grasp65 knockdown suppressed the ability of Fbxw8 knockdown to simplify dendrite arbors and reduce dendrite length in neurons (Figure 7M). Likewise, Grasp65 knockdown suppressed the ability of Cul7 or OBSL1 knockdown to both induce Golgi dispersion and simplify dendrite arbors (Figure S13E–S13H), suggesting that Grasp65 acts downstream of OBSL1 and Cul7 in the regulation of Golgi morphology and dendrite patterning. Collectively, these data reveal that OBSL1, Cul7^Fbxw8^, and Grasp65 are components of a novel ubiquitin ligase pathway that regulates Golgi morphology and dendrite patterning.

## Discussion

We have discovered a Golgi-associated ubiquitin ligase mechanism that plays an essential role in dendrite patterning in the mammalian brain. The ubiquitin ligase Cul7^Fbxw8^ localizes to the Golgi apparatus in mammalian brain neurons. Inhibition of Cul7^Fbxw8^ by independent approaches, including knockdown of Fbxw8, profoundly impairs Golgi structure and function and dramatically inhibits the elaboration and growth of dendrites in primary neurons and in the developing rat cerebellum in vivo. We have identified the cytoskeletal adaptor protein OBSL1 as a key regulator of Cul7^Fbxw8^ that localizes the scaffold protein Cul7 at the Golgi apparatus and thus plays a critical role in Golgi and dendrite morphogenesis. We have also identified the Golgi protein Grasp65 as a novel and physiologically relevant substrate of Cul7^Fbxw8^ in organizing the normal organelle structure of the Golgi complex and for the elaboration of dendrites in neurons. These findings define OBSL1, Cul7^Fbxw8^, and Grasp65 as components of a spatially restricted ubiquitin ligase pathway that governs morphogenesis of the Golgi apparatus and development of dendrites in the brain.

The identification of Cul7^Fbxw8^ as the first ubiquitin ligase that regulates the morphology of the Golgi apparatus in neurons bears significant ramifications for our understanding of the mechanisms that orchestrate dendrite patterning and hence the establishment of neuronal circuitry. Secretory trafficking through the Golgi apparatus is thought to play a particularly important and specific function in the elaboration of dendrites, as dendrites undergo extensive dynamic periods of growth and retraction during development [1],[2]. By controlling Golgi structure and function, Cul7^Fbxw8^ represents a key regulatory mechanism in the organization of Golgi morphology and dendrite morphogenesis. Armed with this function, Cul7^Fbxw8^ may coordinate Golgi morphology in different populations of neurons to achieve the diversity of Golgi morphologies in these neurons and thus give rise to the distinct patterns of dendrite arbors in the brain.

Although ubiquitin ligases have been reported to operate at other subcellular locales, such as the centrosome and nucleus [11],[13], in this study we have uncovered Cul7^Fbxw8^ as the first ubiquitin ligase, to our knowledge, that not only employs the Golgi apparatus as a signaling platform but also orchestrates the morphogenesis and function of the organelle in neurons. Using a proteomics-based screening approach, we have identified OBSL1 as a critical regulator of Cul7^Fbxw8^ in Golgi morphology and dendrite elaboration. OBSL1 is required for localization of Cul7 at the Golgi apparatus, highlighting the importance of subcellular localization for Cul7^Fbxw8^ function in neurons. Remarkably, both OBSL1 and Cul7 are mutated in the inherited human genetic disorder 3M syndrome, characterized by growth retardation [33]–[35]. The genetic relationship between OBSL1 and Cul7 in human disease further strengthens the intimate biochemical link between OBSL1 and Cul7^Fbxw8^ as components of a conserved Golgi-associated ubiquitin ligase pathway. It will be interesting in future studies to investigate the role of deregulation of Golgi morphology and dendrite development in the pathogenesis of 3M syndrome.

We have also uncovered that the Cul7^Fbxw8^-induced ubiquitination and degradation of the major Golgi protein Grasp65 plays a critical role in regulating the structural integrity and function of the Golgi apparatus and dendrite development in neurons. Beyond the control of dendrite patterning in postmitotic neurons, regulation of secretory trafficking plays a crucial role in diverse biological processes from embryogenesis to the establishment of epithelial cell apical-basolateral polarity to cell migration [41],[42]. It will be interesting to determine whether Cul7^Fbxw8^-induced Grasp65 ubiquitination and degradation might also influence Golgi-dependent biological processes in non-neuronal cells.

Grasp65 is a versatile protein with multiple reported functions in the regulation of Golgi architecture. Several studies support a role for Grasp65 in the stacking of Golgi cisternae [43]–[47]. Interestingly, by promoting Golgi cisterna stacking, Grasp65 limits anterograde transport [37]. Grasp65 may also regulate linking of Golgi cisternae [48]. Phosphorylation of Grasp65 regulates Golgi disassembly during mitotic progression in proliferating cells [40],[46],[47],[49]. Finally, Grasp65 is also required for trafficking of a small subset of proteins that contain a C-terminal valine [50]. It will be important to understand the diverse functions of Grasp65 to gain further insights into the role of this major Golgi protein in the structure and function of the Golgi apparatus as well as in dendrite elaboration.

The ubiquitin ligase Cdc20–anaphase promoting complex (Cdc20-APC) functions at the centrosome to promote dendrite development [13]. In future studies, it will be interesting to determine how Cul7^Fbxw8^ signaling at the Golgi apparatus and Cdc20-APC signaling at the centrosome are coordinated in the control of dendrite morphogenesis. In addition to promoting dendrite development, the ubiquitin ligase Cdc20-APC also drives presynaptic differentiation in neurons [51], raising the interesting question of whether Cul7^Fbxw8^ may also have additional roles in neuronal connectivity beyond the morphogenesis of dendrites. It will also be important to determine how OBSL1-regulated Cul7^Fbxw8^ signaling is integrated with other cell-intrinsic regulators of dendrite morphogenesis, including transcription factors, that have been implicated in dendrite patterning [52]–[54].

The expression profile of components of the Cul7^Fbxw8^ ubiquitin signaling pathway in neurons suggests that this pathway is particularly important in establishing the morphology of the Golgi apparatus at a time when neurons form their dendrite arbors during brain development. Since abnormalities in dendrite morphology are thought to play a critical role in neurodevelopmental disorders of cognition, including mental retardation and autism spectrum disorders [55],[56], it will be interesting to explore whether deregulation of Cul7^Fbxw8^ ubiquitin signaling and consequent organelle dysfunction of the Golgi apparatus might contribute to the pathogenesis of these devastating disorders.

## Materials and Methods

### Plasmids

Human Fbxw8 cDNA was cloned into pcDNA3 to generate the Flag-Fbxw8 expression plasmid and into pEGFP-C1 to generate the GFP-Fbxw8 expression plasmid. The Cul7-HA expression plasmid and pBabe/cul7 RNAi plasmid (containing the targeting sequence GTT GAG TAG TCC TGA TTA TCA) were kindly provided by Yang Shi (Harvard Medical School). The following expression plasmids were generously provided: Fbxw8ΔFbox from Keiichi Nakayama (Kyushu University), mCherry-GT from Irina Kaverina (Vanderbilt University), VSVG-ts-GFP from Tom Kirchhausen (Harvard Medical School), V5-OBSL1 from Graeme Black (University of Manchester), Grasp65-GFP from Martin Lowe (University of Manchester), and Grasp55-GFP from Adam Linstedt (Carnegie Mellon University). Deletion mutants of Cul7 and Grasp65 were generated by site-directed mutagenesis (Stratagene) and confirmed by sequencing. The C-terminal region of human Cul7 cDNA starting at base pair A1677 was cloned into pGex4T1 to generate the GST-Cul7C construct. RNAi plasmids were designed as described previously [28], using the following primers: U6/fbxw8-1, 5′-TGA ACA CGA TGC AAG AAT ACA AAG TTA ACG TGT ATT CTT GCA TCG TGT TCA CCC TTT TTG-3′; U6/fbxw8-2, 5′-ATG GAT GAC TGG AAG ATT GTT AAG TTA ACG AAC AAT CTT CCA GTC ATC CAT CCC TTT TTG-3′; U6/obsl1-1, 5′-GTA CGA GCA GAT TGA AGA AAA GTT AAC GTT CTT CAA TCT GCT CGT ACC CCT TTT TG-3′; U6-obsl1-2, 5′-GAG TCA AAT GTG TCA AGC AAA GTT AAC GTG CTT GAC ACA TTT GAC TCC CCT TTT TG-3′; U6/grasp65-1, 5′-AAG GCA CTG CTG AAG GCT AAT AAG TTA ACG ATT AGC CTT CAG CAG TGC CTT CCC TTT TTG-3′; grasp65-2, 5′-GTT CCA GGC AGA GTG ACT ACA GAG TTA ACG TGT AGT CAC TCT GCC TGG AAC CCC TTT TTG-3′. Rescue constructs were generated by engineering silent mutations as follows: Fbxw8, TT**C** GA**G** CA**T** GA**C** GC**T C**G**T** AT**T**; Cul7, G **C**T**C TCC TCC** CC**C** GA**C** TA**C** CA; OBSL1, AA**A** TA**T** GA**A** CA**A** AT**A** GA**G** GA; Grasp65, AA**A** GC**C** CT**C** CT**C** AA**A** GC**C** AA**C**.

### Antibodies

Rabbit Fbxw8 antibodies were kindly provided by James DeCaprio (Harvard Medical School). Rabbit Grasp65 antibodies were kindly provided by Christine Sütterlin (University of California, Irvine). Mouse VSVG antibodies recognizing an external epitope of VSVG were kindly provided by Douglas Lyles (Wake Forest University). Rabbit SnoN, mouse 14-3-3β, mouse Actin, and mouse Hsp60 antibodies were purchased from Santa Cruz Biotechnology. Rabbit GFP, mouse GFP, and mouse V5 antibodies were purchased from Invitrogen, rabbit Erk1/2, from Cell Signaling Technology, mouse TGN38, from Abcam, mouse Flag and mouse Cul7, from Sigma, mouse PDI and mouse GM130, from BD Biosciences, rabbit DsRed, from Clonetech, mouse HA, from Covance, and rat HA-horseradish peroxidase conjugate, from Roche.

### Primary Neuron Culture and Transfection

Primary cerebellar granule neurons were isolated from P6 rat pups and prepared as described [57]. Hippocampal and cortical neurons were isolated from embryonic day 18 rat embryos and prepared as described [58]. Neurons were transfected using a modified calcium phosphate method as described [57]. For high-efficiency transfection, neurons were transfected as indicated with a nucleofection method according to the instructions of the manufacturer (Lonza). In analyses of neuronal morphology, an expression plasmid encoding the anti-apoptotic protein Bcl-X_L_ was included in neuronal transfections to rule out the possibility that the effects of RNAi or protein expression were due to any effect of these manipulations on cell survival. The expression of Bcl-X_L_ has little or no effect on dendrite or axon growth [10],[59],[60]. In analyses of neuron survival, the expression plasmid encoding Bcl-X_L_ was not included in neuronal transfections, and cell death was scored by assessment of the integrity of the nuclei (labeled by the DNA dye bisbenzimide; Hoechst 33258) and of the neuronal processes, as described [61]–[63]. Apoptotic cells harbored pyknotic or fragmented nuclei and disintegrated processes. For analyses of dendrite and Golgi morphology, neurons were transfected at DIV1 or DIV2 and fixed for analysis at DIV5–DIV10 as indicated. For analyses of axon morphology, neurons were transfected 8 h after plating and fixed at DIV3 unless otherwise indicated.

### Immunocytochemistry

Neurons were fixed either in 4% paraformaldehyde (PFA) at room temperature for 10 min or in methanol for 10 min at −20°C, for subcellular localization of endogenous Fbxw8. Following standard protocol, neurons were immunostained with the indicated primary antibodies, followed by fluorescently labeled secondary antibodies (Jackson ImmunoResearch Laboratories).

### Analysis of Neuronal and Golgi Morphology

For neuron morphology, images of transfected granule neurons were captured in a blinded manner using a Nikon eclipse TE2000 epifluorescence microscope at 40× magnification for dendrites and 20× magnification for axons using a digital CCD camera (Diagnostic Instruments). SPOT imaging software was used to quantify the length of axons and dendrites. Specifically, total dendrite length was recorded as the sum of all dendrites per single neuron [59]. For Golgi morphology, neurons were analyzed using a 60× oil immersion objective in a blinded manner. Images were acquired on a Nikon Ti-E microscope equipped with a PerkinElmer UltraVIEW spinning disk confocal system. Images were acquired and analyzed with Volocity software (PerkinElmer). In granule neurons, “dispersed Golgi” was strictly defined as two or more discreet GM130 puncta per transfected neuron. For cortical neurons and hippocampal neurons that have complex Golgi morphology, we defined dispersed Golgi as multiple, discontinuous GM130 puncta.

### Electron Microscopy

For ultrastructural analysis, granule neurons were transfected using a nucleofection method to achieve high transfection efficiency and processed for EM. A Tecnai G^2^ Spirit BioTWIN electron microscope was used for collection of high-magnification EM images.

### In Vivo Electroporation

All experiments using live animals were approved by the Harvard Medical Area Standing Committee on Animals and strictly conformed to their regulatory standards. We performed in vivo electroporation of rat pups as described [10]. Specifically, we injected 10 µg of the indicated plasmid diluted in 0.3% Fast Green and phosphate buffered saline into the cerebellum of each rat pup. Rats were then subjected to three to five electric pulses of 170 mV for 50 ms with 950-ms rest intervals in between. After 5 d, the animals were euthanized. Cerebella were collected, fixed in 4% PFA, incubated in 30% sucrose, sectioned to 40 µm, and subjected to immunohistochemistry following standard protocol. We used the Hoechst nuclear stain to reveal the architecture of the cerebellar cortex and identify the neurons residing in the IGL.

### Membrane Trafficking Assay

For ER to plasma membrane trafficking studies, granule neurons were transfected at DIV2 with an expression plasmid encoding VSVG-ts-GFP and the indicated plasmids. After 3 d, neurons were incubated at 40°C for 8 h to allow for VSVG-ts-GFP retention in the ER. Granule neurons were then either fixed in 4% PFA or incubated at 32°C to permit exit of VSVG-ts-GFP from the ER [29],[30]. After 20, 40, 60, 90, or 120 min had elapsed, granule neurons were fixed in 4% PFA and subjected to immunostaining with an antibody recognizing an extracellular epitope of VSVG prior to permeabilization, and the rabbit GFP antibody after permeabilization. Images were acquired on Nikon a Ti-E microscope equipped with a PerkinElmer UltraVIEW spinning disk confocal system. Images were acquired and analyzed with Volocity software (PerkinElmer). For quantification, neurons were considered to have trafficked VSVG-ts if external VSVG signal was detectable.

### Proteomic Analysis of Fbxw8 Complexes

293T cells expressing HA-Flag-Fbxw8 (∼10^7^ cells) were lysed in a total volume of 4 ml of lysis buffer (50 mM Tris-HCl [pH 7.5], 150 mM NaCl, 0.5% Nonidet P40, Roche complete EDTA-free protease inhibitor cocktail) and processed for proteomic analysis essentially as previously described [31]. Briefly, lysates were subjected to immunoprecipitation with immobilized anti-HA (Sigma) resin (50% slurry), and proteins were eluted with HA-peptide (Sigma). Proteins were precipitated with 20% trichloroacetic acid and the resulting pellet washed once with 10% trichloroacetic acid and four times with cold acetone. Trichloroacetic-acid-precipitated proteins were resuspended in 100 mM ammonium bicarbonate (pH 8.0) with 10% acetonitrile and sequencing grade trypsin (750 ng, Promega), and incubated at 37°C for 4 h. Digested samples were then loaded onto stagetips and washed. Peptides were eluted with 50% acetonitrile/5% formic acid, dried, and resuspended in 10 µl of 5% acetonitrile/5% formic acid. For each LC-MS/MS run using an LTQ linear ion trap mass spectrometer (ThermoFinnigan), 4 µl was loaded onto an 18-cm×125-µm (ID) C18 column, and peptides were eluted using a 50-min 8%–26% acetonitrile gradient. Spectra were acquired using a top-ten method. Each sample was shot twice in succession, followed by a wash with 70% acetonitrile/30% isopropanol. The resulting spectra were searched using Sequest against a target-decoy database of human tryptic peptides. The resulting list of identifications for each was loaded into CompPASS to facilitate a determination of the *D* ^N^ and *Z* scores [31]. Proteins with normalized *D* ^N^ scores greater than 1.0 and *Z* scores greater than 3.5 were considered to be HCIPs.

### Reverse Transcription PCR

Quantitative reverse transcription PCR was performed as previously described [64]. Gene expression was normalized to succinate dehydrogenase levels.

### Biochemical Assays

293T cells and granule neurons were transfected using a DNA calcium phosphate precipitation method or nucleofection method. Cells were lysed in 1% Triton X-100 lysis buffer (with 150 mM sodium chloride, 50 mM Tris [pH 7.5], 2 mM EDTA, and 1 mM DTT, along with an inhibitor cocktail including protease inhibitors PMSF, aprotinin, pepstatin, and leupeptin, and phosphatase inhibitors sodium orthovanadate, sodium fluoride, okadaic acid, and sodium pyrophosphate). Densitometric analysis was performed on scanned autoradiographs using the Image J software (version 1.4). Subcellular fractionation into cytoplasmic fractions and nuclear fractions was performed as previously described [10]. To promote solubilization of ubiquitinated conjugates, 0.1% SDS and 1% sodium deoxycholate were added to immunoprecipitations of Grasp65 proteins using the Grasp65 or GFP antibody. Lysates were pre-cleared with protein A/G sepharose beads prior to immunoprecipitation. An in vitro binding assay using recombinant GST-Cul7C or GST and in vitro transcribed and translated OBSL1 was performed as described previously [63].

## Supporting Information

Figure S1 **Fbxw8 localizes to the Golgi apparatus in granule neurons and hippocampal neurons.** (A) Cerebellar granule neurons were transfected with the expression plasmids encoding GFP-Fbxw8 and mCherry, the latter to visualize neuronal morphology, and subjected to immunocytochemistry with the GFP and DsRed antibodies. A representative image is shown. GFP-Fbxw8 is restricted from axons, dendrites, and the nucleus. Inset shows GFP-Fbxw8 is perinuclear and bean-shaped. Scale bar = 10 µm. (B) Granule neurons were transfected with the GFP-Fbxw8 and mCherry-GT expression plasmids and subjected to immunocytochemistry with the GFP and DsRed antibodies. Hoechst was used to label nuclei. Arrows indicate the perinuclear co-localization of GFP-Fbxw8 with mCherry-GT, which labels the Golgi apparatus. Scale bar = 2 µm. (C) Hippocampal neurons were subjected to immunocytochemistry with the Fbxw8 antibody together with the TGN38 or GM130 antibody. Scale bar = 10 µm. Dotted lines represent tracing of the neuron. (D) Quantification of fold intensity change of Flag-Fbxw8 protein levels normalized to GFP protein levels in 293T cells transfected with U6/fbxw8-1, U6/fbxw8-2, or U6 control RNAi plasmid together with Flag-Fbxw8 and GFP expression plasmids as in Figure 1E. Fbxw8 RNAi significantly reduced Flag-Fbxw8 protein levels (*p<*0.001, ANOVA followed by Bonferroni post hoc test; *n = *3). (E) Quantification of fold intensity change of Fbxw8 protein levels normalized to Erk1/2 protein levels in granule neurons transfected using the nucleofection method with the U6/fbxw8-1, U6/fbxw8-2, or U6 control RNAi plasmid as in Figure 1F. Fbxw8 RNAi significantly reduced Fbxw8 protein levels in granule neurons (*p<*0.05, ANOVA followed by Bonferroni post hoc test; *n = *3).
(TIF)

Figure S2 **Fbxw8 promotes dendrite arborization in granule neurons.** (A) Quantification of dendrite branch number in granule neurons transfected with the U6/fbxw8-1, U6/fbxw8-2, or control U6 RNAi plasmid, analyzed as in Figure 2A, and subjected to morphometric analysis. The number of primary dendrites per neuron was significantly reduced in Fbxw8 knockdown neurons as compared to control U6-transfected neurons (*p<*0.001, ANOVA followed by Bonferroni post hoc test; total neurons measured = 228). (B) The number of secondary and tertiary dendrite branches per neuron was significantly reduced in Fbxw8 knockdown neurons as compared to control U6-transfected neurons (*p<*0.0001, ANOVA followed by Bonferroni post hoc test; total neurons measured = 228). (C) Granule neurons transfected as in Figure 2A and fixed at DIV5, DIV8, or DIV10 were analyzed as in Figure 2B. Quantification revealed that Fbxw8 RNAi significantly reduced dendrite length at each time point (*p<*0.001, ANOVA followed by Bonferroni post hoc test; total neurons measured = 804). (D) Quantification of total axon length of neurons transfected 8 h after plating with the U6/fbxw8-1, U6/fbxw8-2, or control U6 RNAi plasmid, fixed at DIV2, DIV3, or DIV4 and analyzed as in Figure 2D, revealed that Fbxw8 knockdown had little or no effect on axon length at any time point (total neurons measured = 340). (E) Lysates of 293T cells transfected with the Flag-Fbxw8-WT, Flag-Fbxw8-Res, or control vector expression plasmid along with the Fbxw8 RNAi or control U6 RNAi plasmid were immunoblotted with the indicated antibodies. (F) Representative images of granule neurons analyzed as in Figure 2E are shown. Scale bar = 25 µm.
(TIF)

Figure S3 **Fbxw8 promotes dendrite arborization in mammalian brain neurons and in the cerebellar cortex in vivo.** (A) Granule neurons transfected at DIV2 with expression plasmids encoding Fbxw8, Cul7, or control vector together with the GFP expression plasmid were fixed at DIV10 and were subjected to immunocytochemistry with the GFP antibody. Representative images are shown. Scale bar = 25 µm. (B) Quantification of total dendrite length in granule neurons analyzed as in (A). Total dendrite length was significantly increased in Fbxw8- or Cul7- expressing neurons as compared to control neurons (*p<*0.001, ANOVA followed by Bonferroni post hoc test; total neurons measured = 378). (C) Cortical neurons transfected at DIV1 with the U6/fbxw8-1, U6/fbxw8-2, or control U6 RNAi plasmid together with the GFP expression plasmid were fixed at DIV5 and were subjected to immunocytochemistry with the GFP antibody. Morphometric analysis revealed that total dendrite length was significantly reduced in Fbxw8 knockdown cortical neurons as compared to control U6-transfected cortical neurons (*p<*0.001, ANOVA followed by Bonferroni post hoc test; total neurons measured = 259). (D) Hippocampal neurons analyzed as in (C). Morphometric analysis revealed that total dendrite length was significantly reduced in Fbxw8 knockdown hippocampal neurons as compared to control U6-transfected hippocampal neurons (*p<*0.001, ANOVA followed by Bonferroni post hoc test; total neurons measured = 264). (E) Quantification of IGL granule neurons as in Figure 2G revealed that the number of primary dendrites in IGL granule neurons was significantly reduced in Fbxw8 knockdown animals as compared to control animals (*p<*0.001, ANOVA followed by Bonferroni post hoc test; total neurons measured = 234). (F) The number of secondary and tertiary dendrite branches in IGL granule neurons was significantly reduced in Fbxw8 knockdown animals as compared to control animals (*p<*0.001, ANOVA followed by Bonferroni post hoc test; total neurons measured = 234).
(TIF)

Figure S4 **Inhibition of Fbxw8 triggers dispersion of the Golgi apparatus and defects in secretory trafficking in mammalian brain neurons.** (A) Granule neurons were transfected at DIV2 with U6/fbxw8-1 or U6/fbxw8-2 together with the GFP expression plasmid and analyzed as in Figure 3A. Total dendrite length in Fbxw8 knockdown neurons with dispersed Golgi was significantly reduced compared to Fbxw8 knockdown neurons with nondispersed Golgi (*p<*0.005, *t* test; total neurons measured = 159). (B) Cerebral cortical neurons were transfected at DIV1 and analyzed as in Figure 3A. Fbxw8 knockdown significantly increased the percentage of cortical neurons with dispersed Golgi (*p<*0.001, ANOVA followed by Bonferroni post hoc test; *n = *3). (C) Hippocampal neurons were analyzed as in (B). Fbxw8 knockdown significantly increased the percentage of hippocampal neurons with dispersed Golgi (*p<*0.01, ANOVA followed by Bonferroni post hoc test; *n = *3). (D) Granule neurons transfected at DIV2 with U6/fbxw8-1 or U6/fbxw8-2 RNAi plasmid together with the VSVG-ts-GFP expression plasmid were incubated at 40°C at DIV5 for 8 h to allow for ER accumulation and then moved to the trafficking permissive temperature, 32°C, for 2 h as in Figure 3F. Neurons were fixed and subjected to immunocytochemistry using the GFP and GM130 antibodies. In Fbxw8 knockdown neurons in which VSVG-ts did not reach the plasma membrane, VSVG-ts was localized at the Golgi apparatus. Scale bar = 5 µm. (E) Granule neurons analyzed as in Figure 3F were submitted to morphometric analyses. Total dendrite length in Fbxw8 knockdown neurons in which VSVG-ts had trafficked to the plasma membrane and external VSVG-ts was detectable was not significantly different from that of control neurons (total neurons measured = 204).
(TIF)

Figure S5 **Inhibition of Fbxw8 leads to defects in dendritic trafficking.** (A) Granule neurons transfected at DIV2 with the U6/fbxw8-1, U6/fbxw8-2, or control U6 RNAi plasmid together with expression plasmids encoding VSVG-ts-GFP and β-galactosidase, the latter to visualize neuronal morphology, were incubated at 40°C at DIV5 for 8 h to allow for ER accumulation. Neurons were moved to 20°C, which allows for ER to Golgi trafficking but blocks post-Golgi trafficking leading to VSVG-ts accumulation in the Golgi apparatus, and were then fixed after 1 h and subjected to immunocytochemistry with the GFP and β-galactosidase antibodies after permeabilization to visualize Golgi outposts. Representative images are shown. Arrows indicate Golgi outposts. Scale bar = 5 µm. (B) Quantification of granule neurons analyzed as in (A) revealed that the number of Golgi outposts per neuron was significantly reduced in Fbxw8 knockdown neurons as compared to control neurons (*p<*0.001, ANOVA followed by Bonferroni post hoc test; total neurons measured = 261). (C) Granule neurons were transfected at DIV2 with the U6/fbxw8-1, U6/fbxw8-2, or control U6 RNAi plasmid together with expression plasmids encoding GFP-transferrin receptor (TfR) and mCherry. Granule neurons were fixed and subjected to immunocytochemistry with the GFP and DsRed antibodies. Representative images are shown. Arrows indicate dendrites. Scale bar = 10 µm. (D) Quantification of granule neurons analyzed as in (C) revealed that the percentage of cells with GFP-TfR trafficked to dendrites was reduced in Fbxw8 knockdown neurons as compared to control neurons (*p<*0.01, ANOVA followed by Bonferroni post hoc test; *n = *3). (E) Granule neurons transfected with the U6/fbxw8-1 or U6/fbxw8-2 RNAi plasmid together with the GFP-TfR expression plasmid were subjected to immunocytochemistry with the GM130 and GFP antibodies. GFP-TfR and GM130 co-localized in Fbxw8 knockdown neurons. Scale bar = 5 µm. (F) Granule neurons were transfected at DIV2 with the U6/fbxw8-1, U6/fbxw8-2, or control U6 RNAi plasmid together with expression plasmids encoding GFP-synapsin and mCherry. Granule neurons were fixed and subjected to immunocytochemistry with the GFP and DsRed antibodies. Representative images for GFP-synapsin are shown. Arrows indicate GFP-synapsin clusters in the axon. Scale bar = 10 µm. (G) Quantification of granule neurons analyzed as in (F) revealed that the percentage of cells with GFP-synapsin present in the axon was unaffected by Fbxw8 RNAi (*n = *3).
(TIF)

Figure S6 **Cul7 promotes Golgi and dendrite morphogenesis.** (A) Lysates of 293T cells transfected with the Cul7 RNAi plasmid (pBabe/cul7) or the control pBabe plasmid together with the expression plasmids encoding Cul7-HA and GFP were immunoblotted with the HA and GFP antibodies. Cul7 RNAi induced knockdown of exogenous Cul7 in cells. (B) Lysates from granule neurons transfected by nucleofection method with the pBabe/cul7 or control pBabe RNAi plasmid were immunoblotted with the Cul7 and Erk1/2 antibodies. Cul7 RNAi induced knockdown of endogenous Cul7 in granule neurons. (C) Quantification of fold intensity change of Cul7 protein levels normalized to Erk1/2 protein levels in granule neurons transfected with pBabe/cul7 or pBabe control as in (B). Cul7 RNAi significantly reduced Cul7 protein levels in neurons (*p<*0.05, one-sample *t* test; *n = *3). (D) Granule neurons transfected as in Figure 4D were subjected to immunocytochemistry using the GFP and Fbxw8 or TGN38 antibodies as indicated. Representative images are shown. Scale bar = 10 µm. (E) Representative images of granule neurons transfected as in Figure 4J are shown. Scale bar = 25 µm. (F) Granule neurons were transfected at DIV2 with pBabe/cul7 together with the GFP expression plasmid and analyzed as in Figure 3A. Total dendrite length in Cul7 knockdown neurons with dispersed Golgi was significantly reduced compared to that in Cul7 knockdown neurons with nondispersed Golgi (*p<*0.0001, *t* test; total neurons measured = 88). (G) Lysates of Neuro2A cells were immunoprecipitated with the Fbxw8 antibody or pre-immune serum followed by immunoblotting with the Cul7 and Fbxw8 antibodies. Endogenous Cul7 formed a complex with endogenous Fbxw8.
(TIF)

Figure S7 **Inhibition of Cul7 and OBSL1 leads to defects in Golgi integrity in primary neurons and impaired dendrite morphogenesis in the cerebellar cortex in vivo.** (A) Granule neurons transfected by nucleofection method with the pBabe/cul7 or control pBabe RNAi plasmid were collected after 5 d and processed for EM. In control immunocytochemical analyses, we found dispersed Golgi in 59% and 10% of granule neurons transfected with the pBabe/cul7 and pBabe control RNAi plasmid, respectively. A representative EM image is shown for each condition. Scale bar = 500 nm. Right: quantification of cisterna swelling as reflected by measurement of Golgi cisternal thickness. Cul7 knockdown significantly increased Golgi cisternal thickness (*p<*0.05, *t* test; total cisternae measured = 169). (B) Cerebellar sections from P8 rat pups that were electroporated at P3 with the U6/cul7-cmvGFP, U6/obsl1-cmvGFP, or control U6-cmvGFP plasmid were analyzed as in Figure 2F. Representative images of transfected IGL granule neurons are shown for each condition. Scale bar = 25 µm. (C) Quantification of total dendrite length of IGL granule neurons analyzed as in (B). Total dendrite length in IGL granule neurons was significantly decreased in Cul7 and OBSL1 knockdown animals as compared to control animals (*p<*0.001, ANOVA followed by Bonferroni post hoc test; total neurons measured = 275). (D) Quantification of IGL granule neurons as in (B) revealed that the number of primary dendrites in IGL granule neurons was significantly reduced in Cul7 and OBSL1 knockdown animals as compared to control animals (*p<*0.001, ANOVA followed by Bonferroni post hoc test; total neurons measured = 262). (E) The number of secondary and tertiary dendrite branches in IGL granule neurons was significantly reduced in Cul7 and OBSL1 knockdown animals as compared to control animals (*p<*0.01, ANOVA followed by Bonferroni post hoc test; total neurons measured = 265).
(TIF)

Figure S8 **Expression of Fbxw8ΔFbox in neurons triggers defects in Golgi and dendrite morphology.** (A) Granule neurons transfected at DIV2 with an expression plasmid encoding a dominant interfering form of Fbxw8 in which the F-box is deleted (Fbxw8ΔFbox) or the control vector together with the GFP expression plasmid were fixed at DIV5 and were subjected to immunocytochemistry with the GFP and GM130 antibodies. Representative images are shown. Scale bar = 5 µm. (B) Quantification of neurons analyzed as in (A) revealed that the percentage of granule neurons with dispersed Golgi was significantly increased in Fbxw8ΔFbox-expressing neurons as compared to control vector-transfected neurons (*p<*0.01, *t* test; *n = *3). (C) Granule neurons were transfected as in (A) and analyzed as in Figure 2A. Representative images are shown. Scale bar = 25 µm. (D) Morphometric analysis of neurons analyzed as in (C) revealed that total dendrite length was significantly decreased in Fbxw8ΔFbox-expressing neurons as compared to control vector-transfected neurons (*p<*0.0001, *t* test; total neurons measured = 96).
(TIF)

Figure S9 **OBSL1 localizes Cul7 to the Golgi apparatus.** (A) Purified recombinant GST or a GST fusion with a C-terminal domain of Cul7 that included the cullin domain (GST-Cul7C) were incubated with in vitro transcribed and translated V5-OBSL1 and then subjected to pull down using glutathione sepharose followed by immunoblotting with V5 antibodies. GST and GST-Cul7C were visualized using Coomassie Blue stain. (B) OBSL1 mRNA abundance was assessed by quantitative reverse transcription PCR in primary granule neurons at the indicated time points. OBSL1 mRNA abundance decreased with maturation. (C) OBSL1 mRNA abundance was assessed by quantitative reverse transcription PCR from granule neurons transfected by nucleofection method with the U6/obsl1-1, U6/obsl1-2, or U6 control RNAi plasmid. OBSL1 knockdown significantly reduced OBSL1 mRNA levels (*p<*0.001, ANOVA followed by Bonferroni post hoc test; *n = *3). (D) Granule neurons transfected with the U6/obsl1-1, U6/obsl1-2, or U6 control RNAi plasmid together with the Cul7-HA and GFP-GT expression plasmids were subjected to immunocytochemistry using HA and GFP antibodies. Representative images are shown. Scale bar = 10 µm. (E) Lysates of granule neurons transfected by nucleofection method with the U6/obsl1-1, U6/obsl1-2, or U6 control RNAi plasmid were immunoblotted with Cul7 and Akt antibodies. (F) Granule neurons transfected with U6/obsl1-1, U6/obsl1-2, or U6 control plasmid together with the GFP expression plasmid were subjected to immunocytochemistry with Fbxw8 and GFP antibodies. Representative images are shown. Scale bar = 10 µm.
(TIF)

Figure S10 **Localization of OBSL1 and Cul7 to the Golgi apparatus in neurons.** (A) Granule neurons transfected with the U6/fbxw8-1, U6/fbxw8-2, or U6 control RNAi plasmid together with the Cul7-HA and GFP-GT expression plasmids were subjected to immunocytochemistry using HA and GFP antibodies. Representative images are shown. Arrows indicate co-localization of Cul7 with the Golgi apparatus. Scale bar = 10 µm. (B) Granule neurons transfected with expression plasmids encoding Fbxw8ΔFbox or control vector together with the Cul7-HA and GFP-GT expression plasmids were analyzed as in (A). Representative images are shown. Arrows indicate co-localization of Cul7 with the Golgi apparatus. Scale bar = 10 µm. (C) Granule neurons transfected with the U6/fbxw8-1, U6/fbxw8-2, or U6 control RNAi plasmid together with the V5-OBSL1 and GFP-GT expression plasmids were subjected to immunocytochemistry using V5 and GFP antibodies. Representative images are shown. Arrows indicate co-localization of OBSL1 with the Golgi apparatus. Scale bar = 10 µm. (D) Granule neurons transfected with expression plasmids encoding Fbxw8ΔFbox or control vector together with the V5-OBSL1 and GFP-GT expression plasmids were analyzed as in (C). Representative images are shown. Arrows indicate co-localization of OBSL1 with the Golgi apparatus. Scale bar = 10 µm. (E) Granule neurons transfected with the pBabe/cul7 or pBabe control RNAi plasmid together with the V5-OBSL1 and GFP-GT expression plasmids were analyzed as in (C). Representative images are shown. Arrows indicate co-localization of OBSL1 with the Golgi apparatus. Scale bar = 10 µm.
(TIF)

Figure S11 **OBSL1 promotes Golgi and dendrite morphogenesis.** (A) Granule neurons transfected by the nucleofection method with the U6/obsl1-1, U6/obsl1-2, or U6 control RNAi plasmid were collected after 5 d and processed for EM. In control immunocytochemical analyses, we found dispersed Golgi in 58%, 52%, and 8% of granule neurons transfected with the U6/obsl1-1, U6/obsl1-2, and control U6 plasmid, respectively. A representative EM image is shown for each condition. Scale bar = 500 nm. Right: quantification of cisterna swelling as reflected by measurement of Golgi cisternal thickness. OBSL1 knockdown significantly increased Golgi cisternal thickness (*p<*0.05, ANOVA followed by Bonferroni post hoc test; total cisternae measured = 219). (B) Lysates of 293T cells transfected with the V5-OBSL1-WT or V5-OBSL1-Res expression plasmids along with the OBSL1 RNAi or control U6 plasmid were immunoblotted with the indicated antibodies. (C) Granule neurons transfected at DIV2 with the U6/obsl1 or control U6 RNAi plasmid together with the expression plasmid encoding OBSL1-WT, OBSL1-Res, or control vector were analyzed as in Figure 3A. Quantification of percentage neurons with dispersed Golgi revealed that OBSL1-Res but not OBSL1-WT significantly reduced the percent of cells with dispersed Golgi in the background of OBSL1 RNAi (*p<*0.01, ANOVA followed by Bonferroni post hoc test; *n = *3). (D) Granule neurons transfected as in (C) were analyzed as in Figure 2A. Representative images of granule neurons are shown. Scale bar = 25 µm. (E) Quantification of total dendrite length analyzed as in (D) revealed that OBSL1-Res but not OBSL1-WT significantly increased dendrite length in the background of OBSL1 RNAi (*p<*0.01, ANOVA followed by Bonferroni post hoc test; total neurons measured = 360).
(TIF)

Figure S12 **Grasp65 is a ubiquitinated target of Cul7^Fbxw8^ that inhibits dendrite arborization in neurons and in the cerebellum in vivo.** (A) Lysates of granule neurons transfected by nucleofection method with the U6/grasp65-1, U6/grasp65-2, or control U6 RNAi plasmid were immunoblotted with the Grasp65 and Erk1/2 antibodies. Grasp65 RNAi triggered robust knockdown of endogenous Grasp65 in neurons. (B) Quantification of fold protein change of Grasp65 protein levels as compared to Erk1/2 protein levels in granule neurons transfected as in (A). Grasp65 knockdown significantly reduced Grasp65 protein levels in granule neurons (*p<*0.05, ANOVA followed by Bonferroni post hoc test; *n = *3). (C) Granule neurons were transfected at DIV2 with the U6/grasp65-1, U6/grasp65-2, or control U6 RNAi plasmid together with an expression plasmid encoding VSVG-ts-GFP and analyzed as in Figure 3F. Representative images are shown of neurons fixed after 60 min at 32°C. Scale bar = 10 µm. (D) Quantification as in Figure 3G of neurons analyzed as in (C). Best fit curves as determined using a sigmoidal variable slope method are displayed. Grasp65 knockdown significantly increased the percentage of granule neurons with trafficked VSVG-ts at 60 min following ER release (*p<*0.001, two-way ANOVA followed by Bonferroni post hoc test; *n = *3). (E) Quantification of dendrite branch number in granule neurons transfected with the U6/grasp65-1, U6/grasp65-2, or control U6 RNAi plasmid, analyzed as in Figure 7A, and subjected to morphometric analysis. The number of primary dendrites per neuron was significantly increased in Grasp65 knockdown neurons as compared to control U6-transfected neurons (*p<*0.001, ANOVA followed by Bonferroni post hoc test; total neurons measured = 294). (F) The number of secondary and tertiary dendrite branches per neuron was significantly increased in Grasp65 knockdown neurons as compared to control U6-transfected neurons (*p<*0.01, ANOVA followed by Bonferroni post hoc test; total neurons measured = 294). (G) Quantification of IGL granule neuron dendrite branching in the cerebellar cortex in P8 rat pups that were subjected to electroporation as described in Figure 7E. The number of primary dendrites in IGL granule neurons was significantly increased in Grasp65 knockdown animals as compared to control animals (*p<*0.0001, *t* test; total neurons measured = 110). (H) The number of secondary and tertiary dendrite branches in IGL granule neurons was significantly increased in Grasp65 knockdown animals as compared to control animals (*p<*0.0005, *t* test; total neurons measured = 110).
(TIF)

Figure S13 **Grasp65 operates downstream of OBSL1 and Cul7^Fbxw8^ in the control of Golgi and dendrite morphogenesis.** (A) Lysates of 293T cells transfected with the Flag-Fbxw8 expression plasmid together with the Grasp65-GFP or Grasp55-GFP expression plasmid or their control vector were immunoprecipitated with the GFP antibody. Immunoprecipitates and lysates were immunoblotted with the indicated antibodies. (B) Lysates of 293T cells transfected with the expression plasmids encoding HA-ubiquitin, Cul7, and Flag-Fbxw8 or control vector together with Grasp65-GFP or Grasp65ΔPDZ1 were incubated in 2% SDS and then diluted 1∶20 in normal lysis buffer prior to immunoprecipitation with GFP antibodies. Immunoprecipitates were immunoblotted with the HA and GFP antibodies. Fbxw8 triggered the accumulation of polyubiquitinated Grasp65 but not Grasp65ΔPDZ1 in cells. (C) Lysates of 293T cells transfected with expression plasmids encoding Grasp65-GFP or Grasp65ΔPDZ1 together with Flag-Fbxw8, a mutant lacking the F-box domain (ΔFbox), or the control vector were immunoblotted with the indicated antibodies. Fbxw8 triggered the downregulation of Grasp65 but not Grasp65ΔPDZ1 in cells. (D) Quantification of fold intensity change of Grasp65 protein levels as compared to Actin protein levels in granule neurons transfected as in Figure 7K. Fbxw8 knockdown significantly increased Grasp65 protein levels in granule neurons (*p<*0.05, ANOVA followed by Bonferroni post hoc test; *n = *4). (E) Granule neurons transfected with the pBabe/cul7, U6/grasp65, or control pBabe or U6 plasmids, separately or in combination, were fixed and analyzed as in Figure 3A. Grasp65 knockdown significantly suppressed Cul7 knockdown-induced Golgi dispersion (*p<*0.001, ANOVA followed by Bonferroni post hoc test; *n = *3). (F) Granule neurons transfected as in (E) and analyzed as in Figure 2A. Grasp65 knockdown significantly suppressed Cul7 knockdown-induced reduction in dendrite length (*p<*0.001, ANOVA followed by Bonferroni post hoc test; total neurons measured = 488). (G) Granule neurons transfected with the U6/obsl1, U6/grasp65, or control U6 plasmid, separately or in combination, were fixed and analyzed as in Figure 3A. Grasp65 knockdown significantly suppressed OBSL1 knockdown-induced Golgi dispersion (*p<*0.05, ANOVA followed by Bonferroni post hoc test; *n = *3). (H) Granule neurons transfected as in (G) and analyzed as in Figure 2A. Grasp65 knockdown significantly suppressed OBSL1 knockdown-induced reduction in dendrite length (*p<*0.01, ANOVA followed by Bonferroni post hoc test; total neurons measured = 350).
(TIF)

## Abbreviations

DIV[number]: day in vitro [number]
EM: electron microscopy
ER: endoplasmic reticulum
GFP: green fluorescent protein
GST: glutathione S-transferase
HCIP: high-confidence candidate-interacting protein
IGL: internal granule layer
IP/mass spec: immunoprecipitation/mass spectrometry
LC-MS/MS: liquid chromatogrphy–tandem mass spectrometry
P[number]
postnatal day [number]
PFA: paraformaldehyde
RNAi: RNA interference
