# Supplementary figures and images for: An OBSL1-Cul7Fbxw8 Ubiquitin Ligase Signaling Mechanism Regulates Golgi Morphology and Dendrite Patterning
Source: PLoS Biol. 2011 May 10;9(5):e1001060. doi: 10.1371/journal.pbio.1001060 (PMC3091842; doi:10.1371/journal.pbio.1001060)

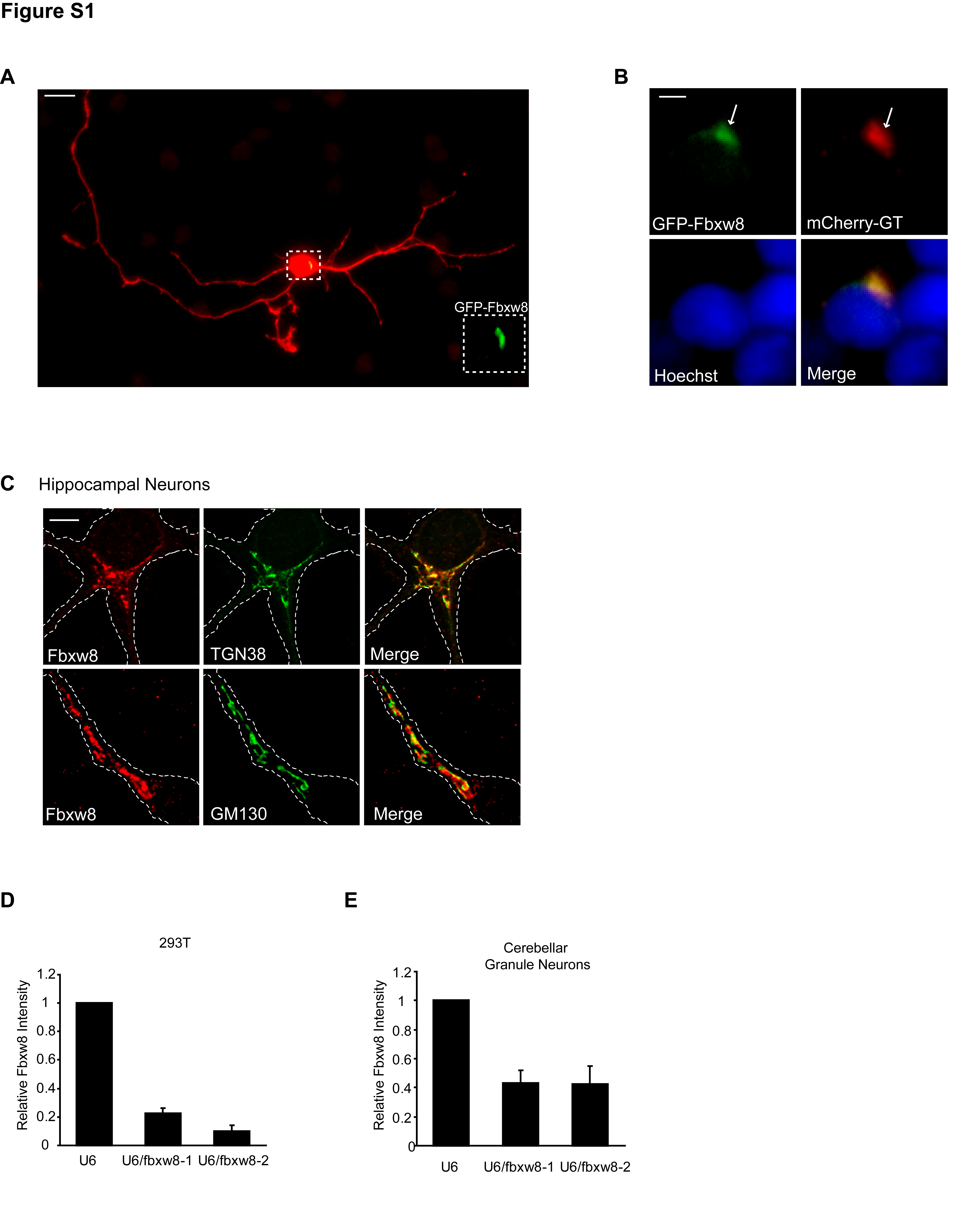

Supplement: Figure S1 — Fbxw8 localizes to the Golgi apparatus in granule neurons and hippocampal neurons. (A) Cerebellar granule neurons were transfected with the expression plasmids encoding GFP-Fbxw8 and mCherry, the latter to visualize neuronal morphology, and subjected to immunocytochemistry with the GFP and DsRed antibodies. A representative image is shown. GFP-Fbxw8 is restricted from axons, dendrites, and the nucleus. Inset shows GFP-Fbxw8 is perinuclear and bean-shaped. Scale bar = 10 µm. (B) Granule neurons were transfected with the GFP-Fbxw8 and mCherry-GT expression plasmids and subjected to immunocytochemistry with the GFP and DsRed antibodies. Hoechst was used to label nuclei. Arrows indicate the perinuclear co-localization of GFP-Fbxw8 with mCherry-GT, which labels the Golgi apparatus. Scale bar = 2 µm. (C) Hippocampal neurons were subjected to immunocytochemistry with the Fbxw8 antibody together with the TGN38 or GM130 antibody. Scale bar = 10 µm. Dotted lines represent tracing of the neuron. (D) Quantification of fold intensity change of Flag-Fbxw8 protein levels normalized to GFP protein levels in 293T cells transfected with U6/fbxw8-1, U6/fbxw8-2, or U6 control RNAi plasmid together with Flag-Fbxw8 and GFP expression plasmids as in Figure 1E. Fbxw8 RNAi significantly reduced Flag-Fbxw8 protein levels (p<0.001, ANOVA followed by Bonferroni post hoc test; n = 3). (E) Quantification of fold intensity change of Fbxw8 protein levels normalized to Erk1/2 protein levels in granule neurons transfected using the nucleofection method with the U6/fbxw8-1, U6/fbxw8-2, or U6 control RNAi plasmid as in Figure 1F. Fbxw8 RNAi significantly reduced Fbxw8 protein levels in granule neurons (p<0.05, ANOVA followed by Bonferroni post hoc test; n = 3). (TIF) [file pbio.1001060.s001.tif]

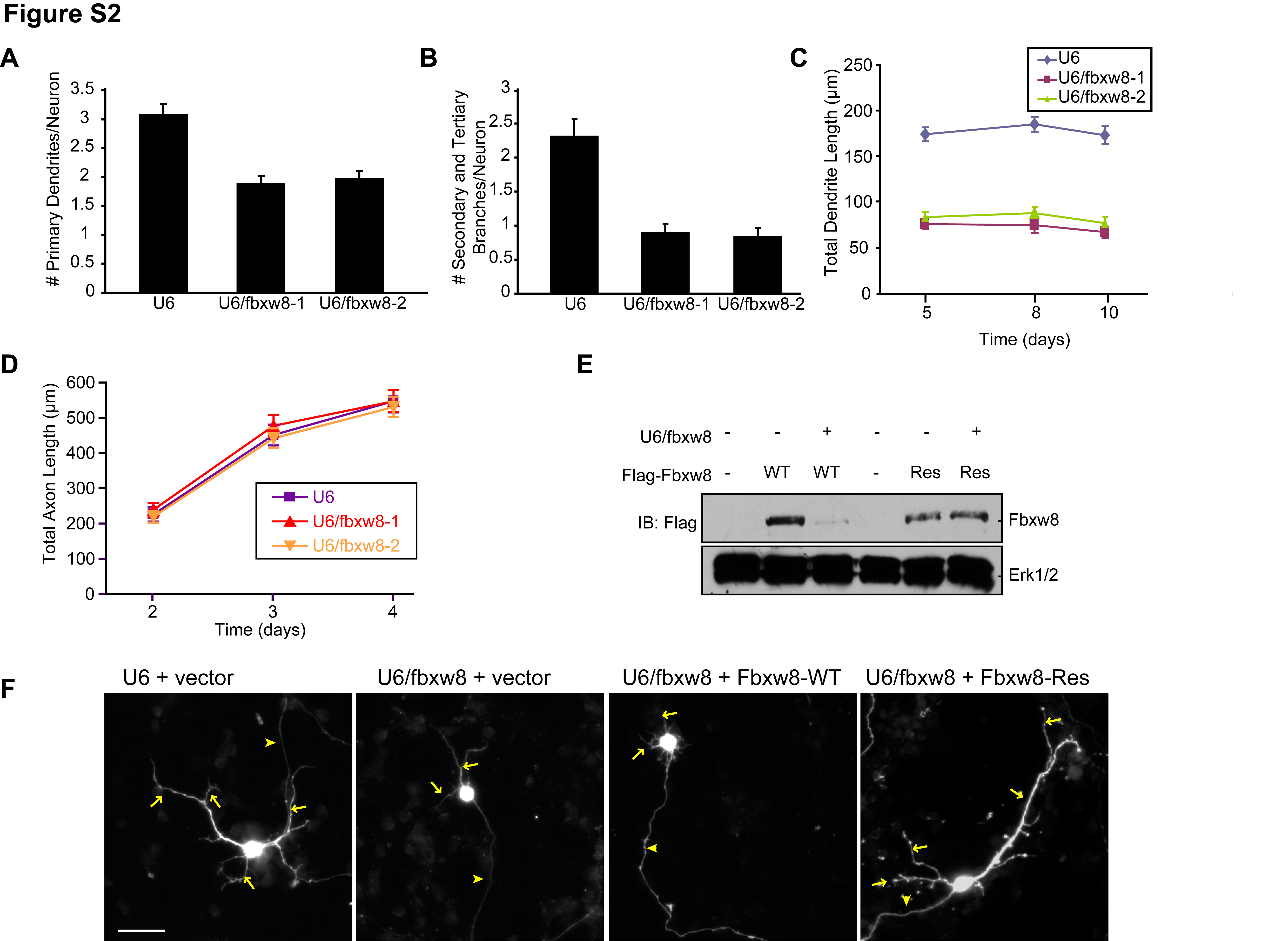

Supplement: Figure S2 — Fbxw8 promotes dendrite arborization in granule neurons. (A) Quantification of dendrite branch number in granule neurons transfected with the U6/fbxw8-1, U6/fbxw8-2, or control U6 RNAi plasmid, analyzed as in Figure 2A, and subjected to morphometric analysis. The number of primary dendrites per neuron was significantly reduced in Fbxw8 knockdown neurons as compared to control U6-transfected neurons (p<0.001, ANOVA followed by Bonferroni post hoc test; total neurons measured = 228). (B) The number of secondary and tertiary dendrite branches per neuron was significantly reduced in Fbxw8 knockdown neurons as compared to control U6-transfected neurons (p<0.0001, ANOVA followed by Bonferroni post hoc test; total neurons measured = 228). (C) Granule neurons transfected as in Figure 2A and fixed at DIV5, DIV8, or DIV10 were analyzed as in Figure 2B. Quantification revealed that Fbxw8 RNAi significantly reduced dendrite length at each time point (p<0.001, ANOVA followed by Bonferroni post hoc test; total neurons measured = 804). (D) Quantification of total axon length of neurons transfected 8 h after plating with the U6/fbxw8-1, U6/fbxw8-2, or control U6 RNAi plasmid, fixed at DIV2, DIV3, or DIV4 and analyzed as in Figure 2D, revealed that Fbxw8 knockdown had little or no effect on axon length at any time point (total neurons measured = 340). (E) Lysates of 293T cells transfected with the Flag-Fbxw8-WT, Flag-Fbxw8-Res, or control vector expression plasmid along with the Fbxw8 RNAi or control U6 RNAi plasmid were immunoblotted with the indicated antibodies. (F) Representative images of granule neurons analyzed as in Figure 2E are shown. Scale bar = 25 µm. (TIF) [file pbio.1001060.s002.tif]

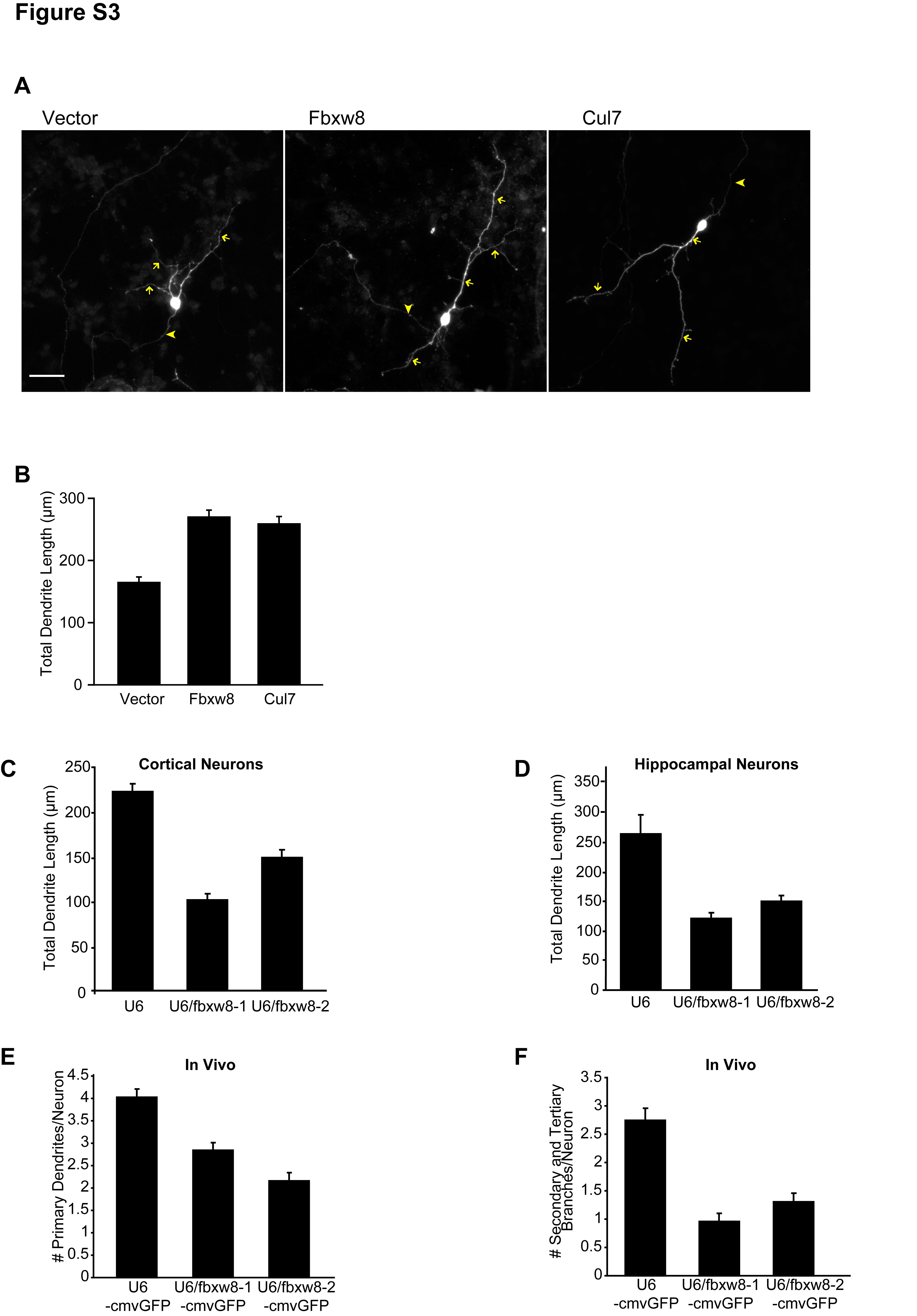

Supplement: Figure S3 — Fbxw8 promotes dendrite arborization in mammalian brain neurons and in the cerebellar cortex in vivo. (A) Granule neurons transfected at DIV2 with expression plasmids encoding Fbxw8, Cul7, or control vector together with the GFP expression plasmid were fixed at DIV10 and were subjected to immunocytochemistry with the GFP antibody. Representative images are shown. Scale bar = 25 µm. (B) Quantification of total dendrite length in granule neurons analyzed as in (A). Total dendrite length was significantly increased in Fbxw8- or Cul7- expressing neurons as compared to control neurons (p<0.001, ANOVA followed by Bonferroni post hoc test; total neurons measured = 378). (C) Cortical neurons transfected at DIV1 with the U6/fbxw8-1, U6/fbxw8-2, or control U6 RNAi plasmid together with the GFP expression plasmid were fixed at DIV5 and were subjected to immunocytochemistry with the GFP antibody. Morphometric analysis revealed that total dendrite length was significantly reduced in Fbxw8 knockdown cortical neurons as compared to control U6-transfected cortical neurons (p<0.001, ANOVA followed by Bonferroni post hoc test; total neurons measured = 259). (D) Hippocampal neurons analyzed as in (C). Morphometric analysis revealed that total dendrite length was significantly reduced in Fbxw8 knockdown hippocampal neurons as compared to control U6-transfected hippocampal neurons (p<0.001, ANOVA followed by Bonferroni post hoc test; total neurons measured = 264). (E) Quantification of IGL granule neurons as in Figure 2G revealed that the number of primary dendrites in IGL granule neurons was significantly reduced in Fbxw8 knockdown animals as compared to control animals (p<0.001, ANOVA followed by Bonferroni post hoc test; total neurons measured = 234). (F) The number of secondary and tertiary dendrite branches in IGL granule neurons was significantly reduced in Fbxw8 knockdown animals as compared to control animals (p<0.001, ANOVA followed by Bonferroni post hoc test; total neurons me [file pbio.1001060.s003.tif]

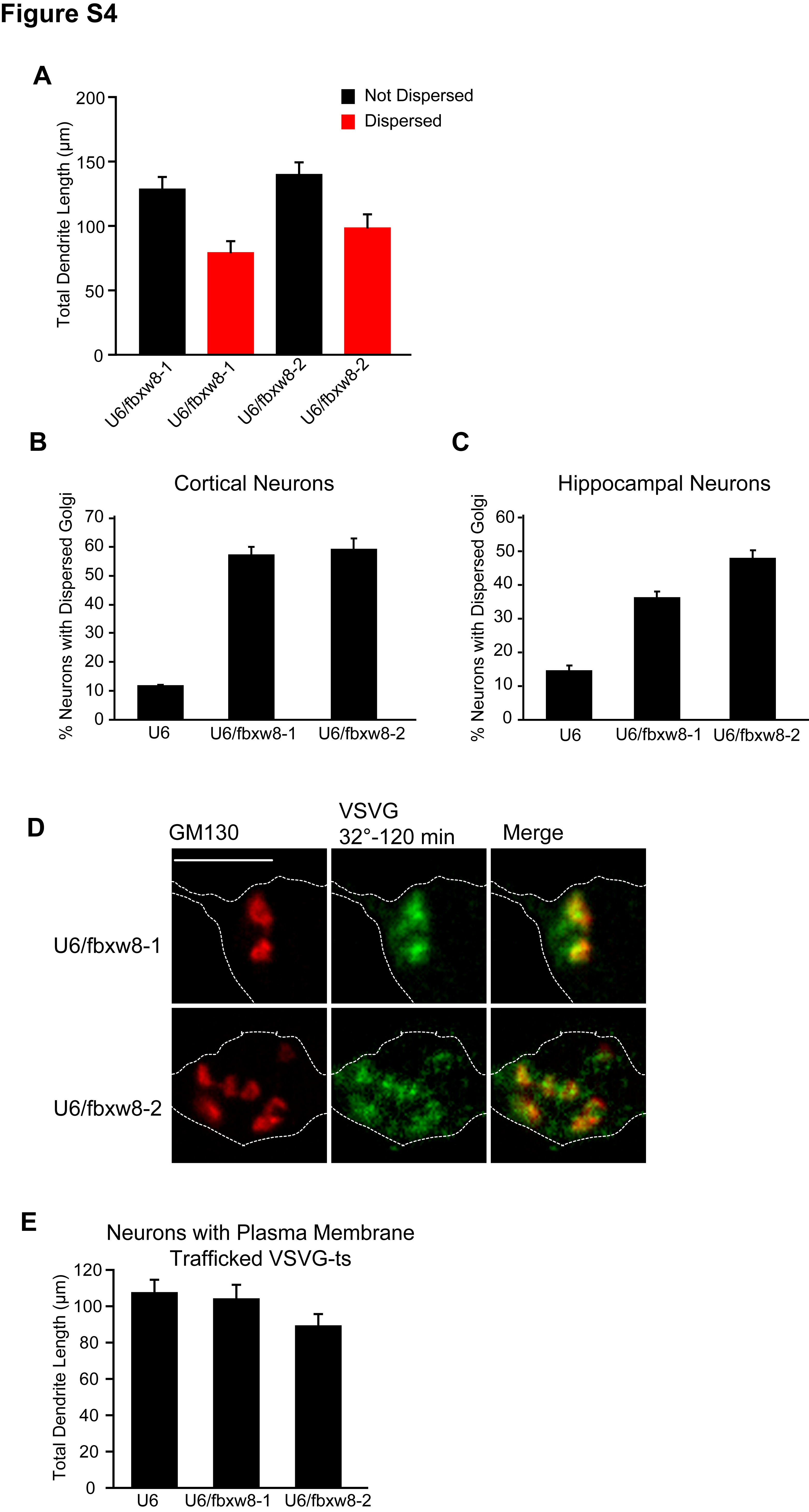

Supplement: Figure S4 — Inhibition of Fbxw8 triggers dispersion of the Golgi apparatus and defects in secretory trafficking in mammalian brain neurons. (A) Granule neurons were transfected at DIV2 with U6/fbxw8-1 or U6/fbxw8-2 together with the GFP expression plasmid and analyzed as in Figure 3A. Total dendrite length in Fbxw8 knockdown neurons with dispersed Golgi was significantly reduced compared to Fbxw8 knockdown neurons with nondispersed Golgi (p<0.005, t test; total neurons measured = 159). (B) Cerebral cortical neurons were transfected at DIV1 and analyzed as in Figure 3A. Fbxw8 knockdown significantly increased the percentage of cortical neurons with dispersed Golgi (p<0.001, ANOVA followed by Bonferroni post hoc test; n = 3). (C) Hippocampal neurons were analyzed as in (B). Fbxw8 knockdown significantly increased the percentage of hippocampal neurons with dispersed Golgi (p<0.01, ANOVA followed by Bonferroni post hoc test; n = 3). (D) Granule neurons transfected at DIV2 with U6/fbxw8-1 or U6/fbxw8-2 RNAi plasmid together with the VSVG-ts-GFP expression plasmid were incubated at 40°C at DIV5 for 8 h to allow for ER accumulation and then moved to the trafficking permissive temperature, 32°C, for 2 h as in Figure 3F. Neurons were fixed and subjected to immunocytochemistry using the GFP and GM130 antibodies. In Fbxw8 knockdown neurons in which VSVG-ts did not reach the plasma membrane, VSVG-ts was localized at the Golgi apparatus. Scale bar = 5 µm. (E) Granule neurons analyzed as in Figure 3F were submitted to morphometric analyses. Total dendrite length in Fbxw8 knockdown neurons in which VSVG-ts had trafficked to the plasma membrane and external VSVG-ts was detectable was not significantly different from that of control neurons (total neurons measured = 204). (TIF) [file pbio.1001060.s004.tif]

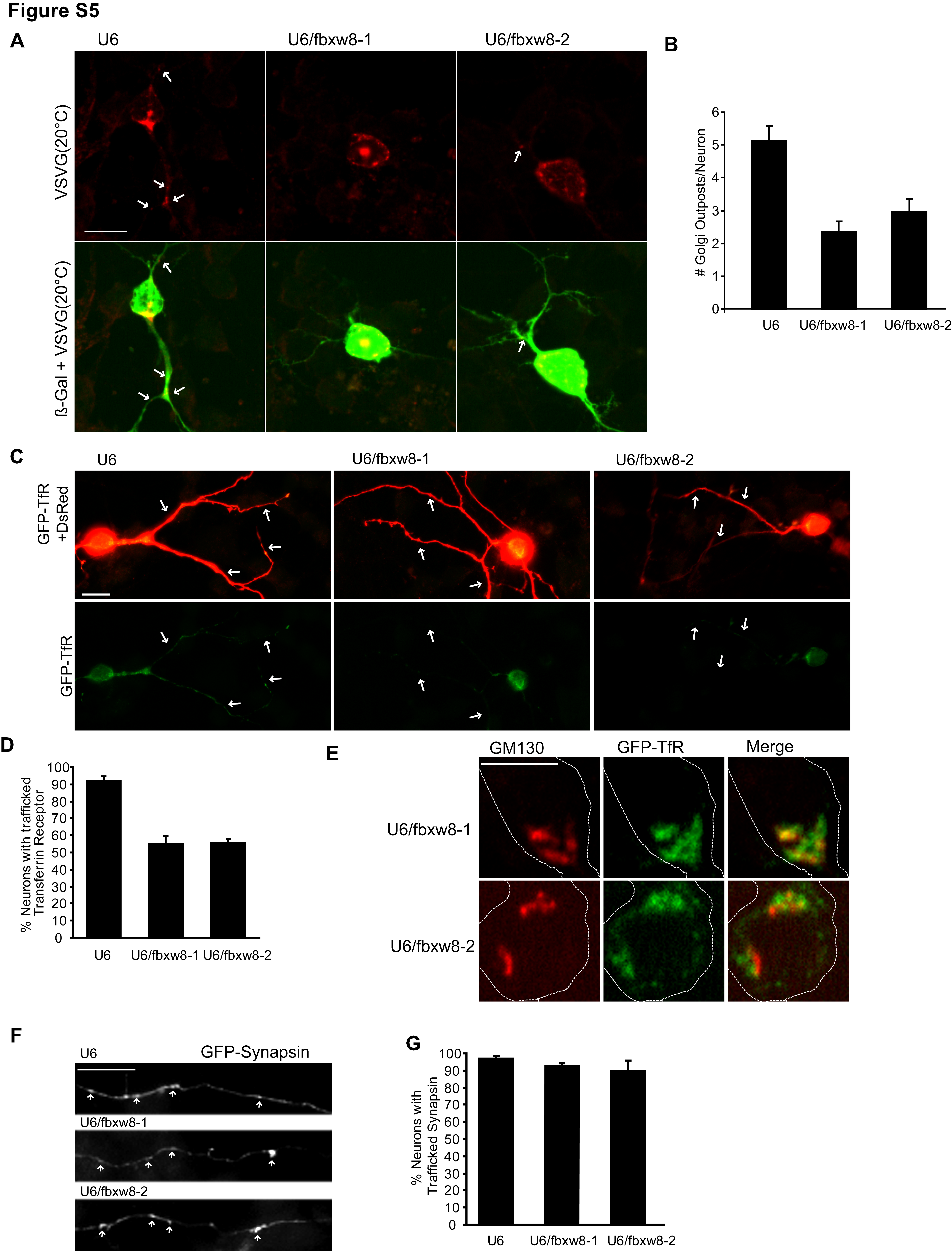

Supplement: Figure S5 — Inhibition of Fbxw8 leads to defects in dendritic trafficking. (A) Granule neurons transfected at DIV2 with the U6/fbxw8-1, U6/fbxw8-2, or control U6 RNAi plasmid together with expression plasmids encoding VSVG-ts-GFP and β-galactosidase, the latter to visualize neuronal morphology, were incubated at 40°C at DIV5 for 8 h to allow for ER accumulation. Neurons were moved to 20°C, which allows for ER to Golgi trafficking but blocks post-Golgi trafficking leading to VSVG-ts accumulation in the Golgi apparatus, and were then fixed after 1 h and subjected to immunocytochemistry with the GFP and β-galactosidase antibodies after permeabilization to visualize Golgi outposts. Representative images are shown. Arrows indicate Golgi outposts. Scale bar = 5 µm. (B) Quantification of granule neurons analyzed as in (A) revealed that the number of Golgi outposts per neuron was significantly reduced in Fbxw8 knockdown neurons as compared to control neurons (p<0.001, ANOVA followed by Bonferroni post hoc test; total neurons measured = 261). (C) Granule neurons were transfected at DIV2 with the U6/fbxw8-1, U6/fbxw8-2, or control U6 RNAi plasmid together with expression plasmids encoding GFP-transferrin receptor (TfR) and mCherry. Granule neurons were fixed and subjected to immunocytochemistry with the GFP and DsRed antibodies. Representative images are shown. Arrows indicate dendrites. Scale bar = 10 µm. (D) Quantification of granule neurons analyzed as in (C) revealed that the percentage of cells with GFP-TfR trafficked to dendrites was reduced in Fbxw8 knockdown neurons as compared to control neurons (p<0.01, ANOVA followed by Bonferroni post hoc test; n = 3). (E) Granule neurons transfected with the U6/fbxw8-1 or U6/fbxw8-2 RNAi plasmid together with the GFP-TfR expression plasmid were subjected to immunocytochemistry with the GM130 and GFP antibodies. GFP-TfR and GM130 co-localized in Fbxw8 knockdown neurons. Scale bar = 5 µm. (F) Granule neurons were transfected at DIV2 with the U [file pbio.1001060.s005.tif]

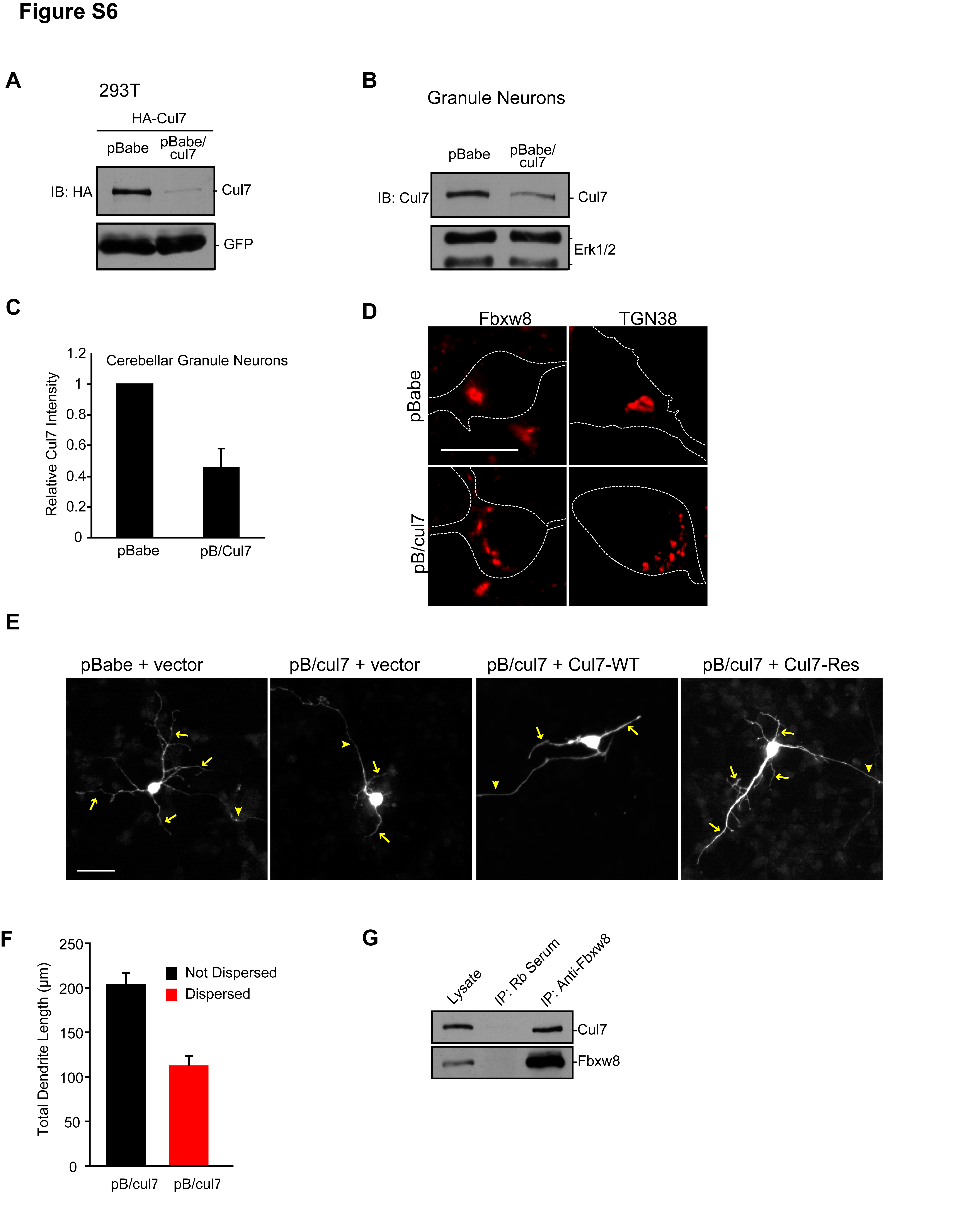

Supplement: Figure S6 — Cul7 promotes Golgi and dendrite morphogenesis. (A) Lysates of 293T cells transfected with the Cul7 RNAi plasmid (pBabe/cul7) or the control pBabe plasmid together with the expression plasmids encoding Cul7-HA and GFP were immunoblotted with the HA and GFP antibodies. Cul7 RNAi induced knockdown of exogenous Cul7 in cells. (B) Lysates from granule neurons transfected by nucleofection method with the pBabe/cul7 or control pBabe RNAi plasmid were immunoblotted with the Cul7 and Erk1/2 antibodies. Cul7 RNAi induced knockdown of endogenous Cul7 in granule neurons. (C) Quantification of fold intensity change of Cul7 protein levels normalized to Erk1/2 protein levels in granule neurons transfected with pBabe/cul7 or pBabe control as in (B). Cul7 RNAi significantly reduced Cul7 protein levels in neurons (p<0.05, one-sample t test; n = 3). (D) Granule neurons transfected as in Figure 4D were subjected to immunocytochemistry using the GFP and Fbxw8 or TGN38 antibodies as indicated. Representative images are shown. Scale bar = 10 µm. (E) Representative images of granule neurons transfected as in Figure 4J are shown. Scale bar = 25 µm. (F) Granule neurons were transfected at DIV2 with pBabe/cul7 together with the GFP expression plasmid and analyzed as in Figure 3A. Total dendrite length in Cul7 knockdown neurons with dispersed Golgi was significantly reduced compared to that in Cul7 knockdown neurons with nondispersed Golgi (p<0.0001, t test; total neurons measured = 88). (G) Lysates of Neuro2A cells were immunoprecipitated with the Fbxw8 antibody or pre-immune serum followed by immunoblotting with the Cul7 and Fbxw8 antibodies. Endogenous Cul7 formed a complex with endogenous Fbxw8. (TIF) [file pbio.1001060.s006.tif]

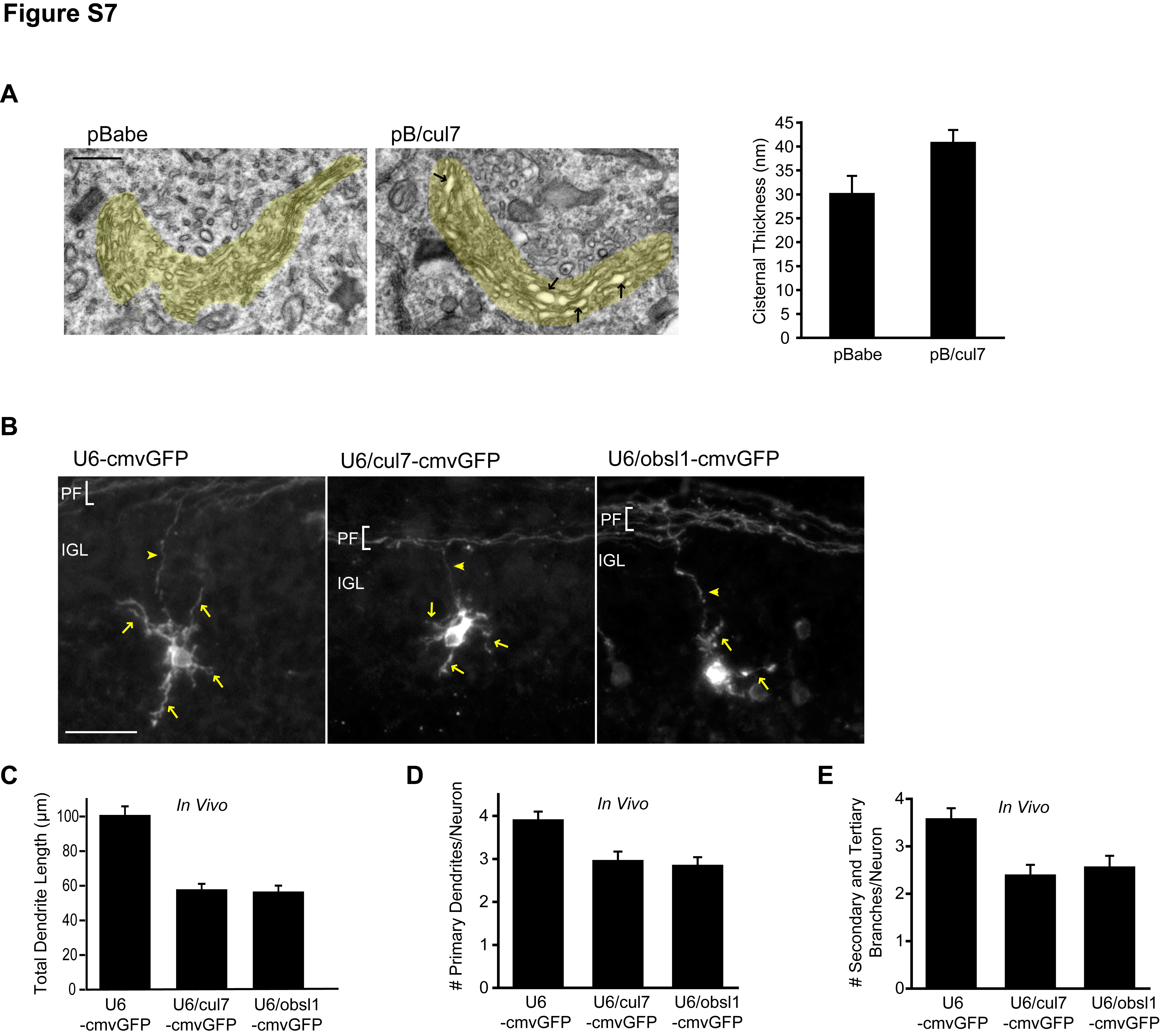

Supplement: Figure S7 — Inhibition of Cul7 and OBSL1 leads to defects in Golgi integrity in primary neurons and impaired dendrite morphogenesis in the cerebellar cortex in vivo. (A) Granule neurons transfected by nucleofection method with the pBabe/cul7 or control pBabe RNAi plasmid were collected after 5 d and processed for EM. In control immunocytochemical analyses, we found dispersed Golgi in 59% and 10% of granule neurons transfected with the pBabe/cul7 and pBabe control RNAi plasmid, respectively. A representative EM image is shown for each condition. Scale bar = 500 nm. Right: quantification of cisterna swelling as reflected by measurement of Golgi cisternal thickness. Cul7 knockdown significantly increased Golgi cisternal thickness (p<0.05, t test; total cisternae measured = 169). (B) Cerebellar sections from P8 rat pups that were electroporated at P3 with the U6/cul7-cmvGFP, U6/obsl1-cmvGFP, or control U6-cmvGFP plasmid were analyzed as in Figure 2F. Representative images of transfected IGL granule neurons are shown for each condition. Scale bar = 25 µm. (C) Quantification of total dendrite length of IGL granule neurons analyzed as in (B). Total dendrite length in IGL granule neurons was significantly decreased in Cul7 and OBSL1 knockdown animals as compared to control animals (p<0.001, ANOVA followed by Bonferroni post hoc test; total neurons measured = 275). (D) Quantification of IGL granule neurons as in (B) revealed that the number of primary dendrites in IGL granule neurons was significantly reduced in Cul7 and OBSL1 knockdown animals as compared to control animals (p<0.001, ANOVA followed by Bonferroni post hoc test; total neurons measured = 262). (E) The number of secondary and tertiary dendrite branches in IGL granule neurons was significantly reduced in Cul7 and OBSL1 knockdown animals as compared to control animals (p<0.01, ANOVA followed by Bonferroni post hoc test; total neurons measured = 265). (TIF) [file pbio.1001060.s007.tif]

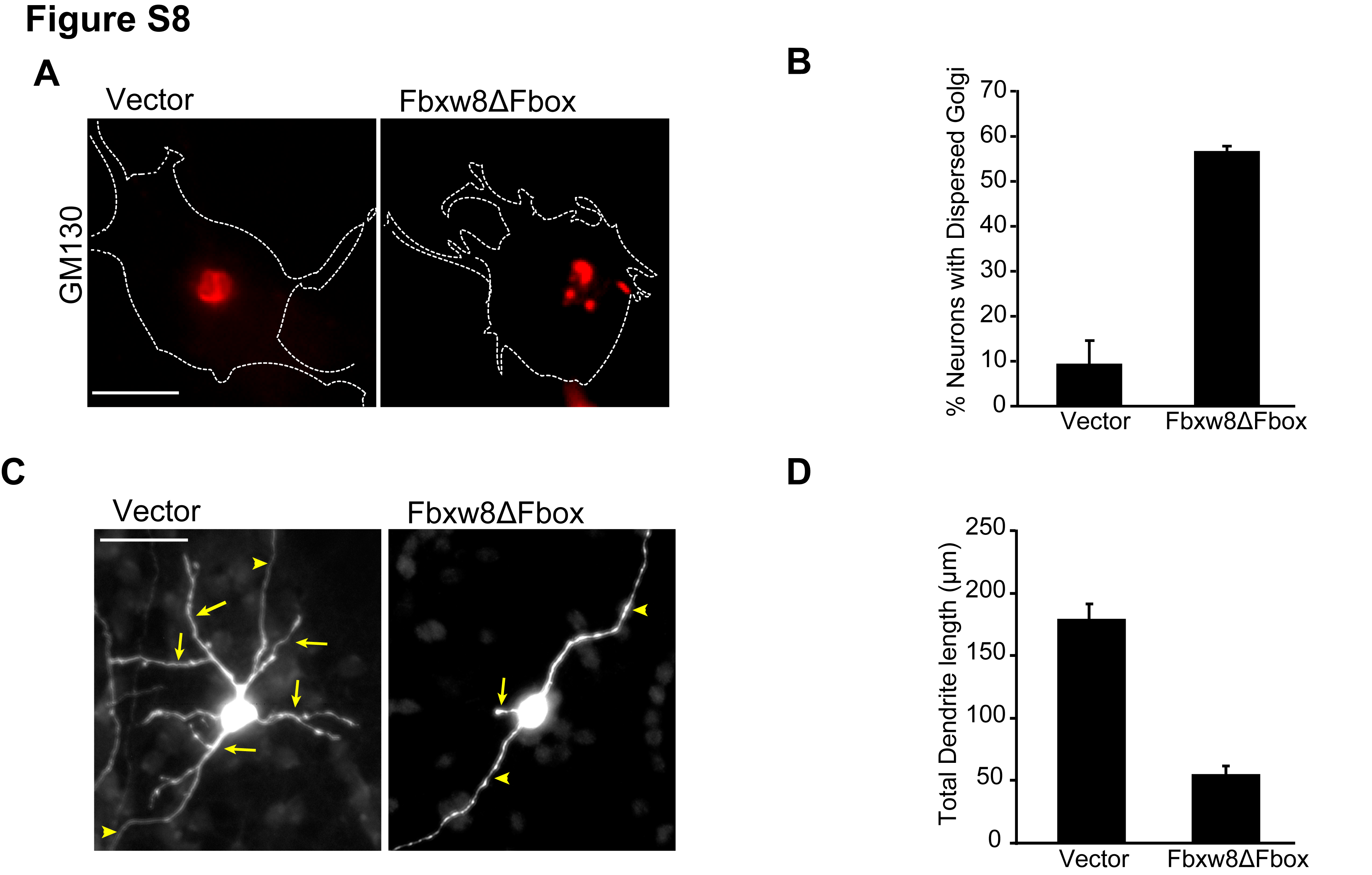

Supplement: Figure S8 — Expression of Fbxw8ΔFbox in neurons triggers defects in Golgi and dendrite morphology. (A) Granule neurons transfected at DIV2 with an expression plasmid encoding a dominant interfering form of Fbxw8 in which the F-box is deleted (Fbxw8ΔFbox) or the control vector together with the GFP expression plasmid were fixed at DIV5 and were subjected to immunocytochemistry with the GFP and GM130 antibodies. Representative images are shown. Scale bar = 5 µm. (B) Quantification of neurons analyzed as in (A) revealed that the percentage of granule neurons with dispersed Golgi was significantly increased in Fbxw8ΔFbox-expressing neurons as compared to control vector-transfected neurons (p<0.01, t test; n = 3). (C) Granule neurons were transfected as in (A) and analyzed as in Figure 2A. Representative images are shown. Scale bar = 25 µm. (D) Morphometric analysis of neurons analyzed as in (C) revealed that total dendrite length was significantly decreased in Fbxw8ΔFbox-expressing neurons as compared to control vector-transfected neurons (p<0.0001, t test; total neurons measured = 96). (TIF) [file pbio.1001060.s008.tif]

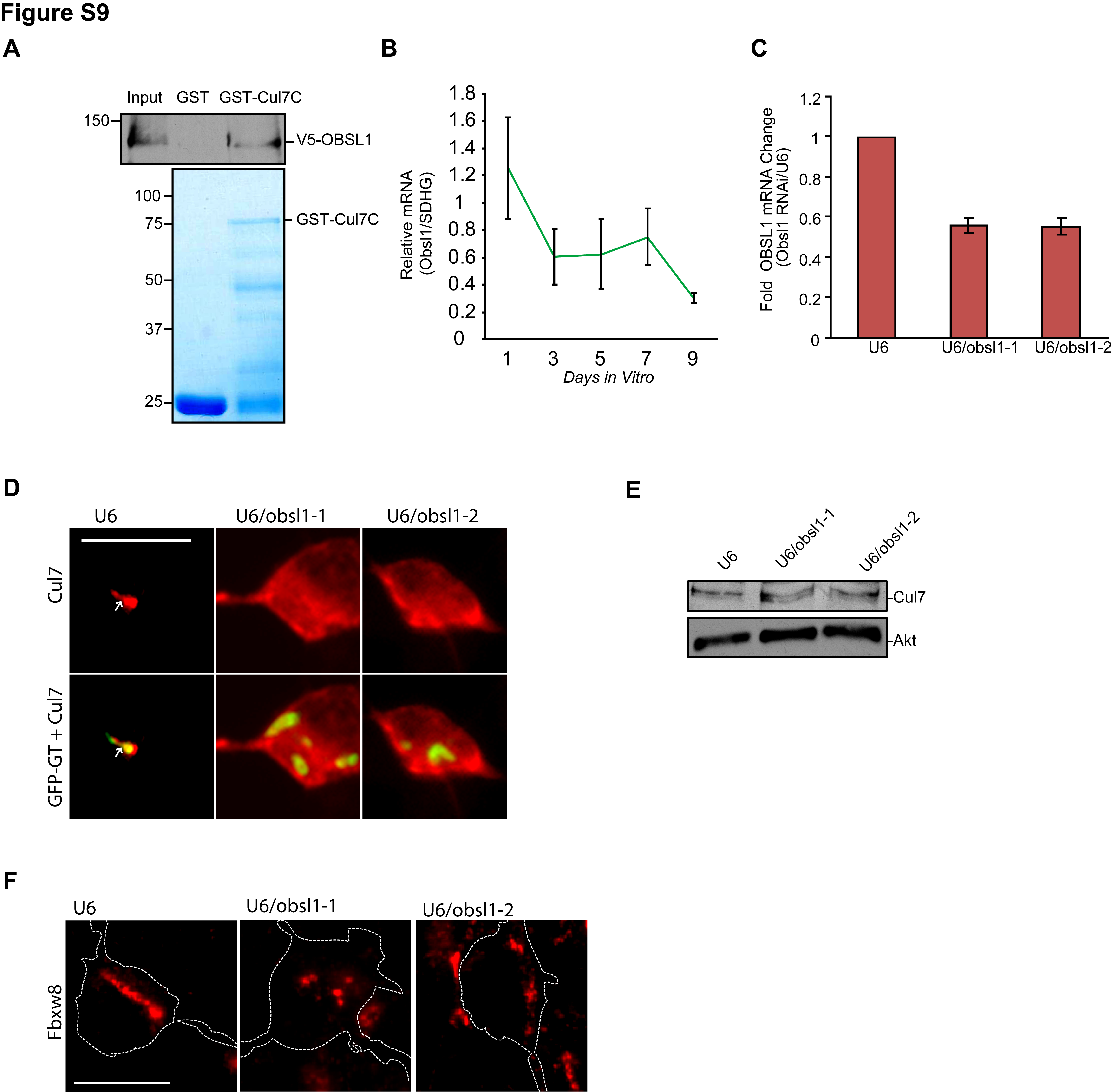

Supplement: Figure S9 — OBSL1 localizes Cul7 to the Golgi apparatus. (A) Purified recombinant GST or a GST fusion with a C-terminal domain of Cul7 that included the cullin domain (GST-Cul7C) were incubated with in vitro transcribed and translated V5-OBSL1 and then subjected to pull down using glutathione sepharose followed by immunoblotting with V5 antibodies. GST and GST-Cul7C were visualized using Coomassie Blue stain. (B) OBSL1 mRNA abundance was assessed by quantitative reverse transcription PCR in primary granule neurons at the indicated time points. OBSL1 mRNA abundance decreased with maturation. (C) OBSL1 mRNA abundance was assessed by quantitative reverse transcription PCR from granule neurons transfected by nucleofection method with the U6/obsl1-1, U6/obsl1-2, or U6 control RNAi plasmid. OBSL1 knockdown significantly reduced OBSL1 mRNA levels (p<0.001, ANOVA followed by Bonferroni post hoc test; n = 3). (D) Granule neurons transfected with the U6/obsl1-1, U6/obsl1-2, or U6 control RNAi plasmid together with the Cul7-HA and GFP-GT expression plasmids were subjected to immunocytochemistry using HA and GFP antibodies. Representative images are shown. Scale bar = 10 µm. (E) Lysates of granule neurons transfected by nucleofection method with the U6/obsl1-1, U6/obsl1-2, or U6 control RNAi plasmid were immunoblotted with Cul7 and Akt antibodies. (F) Granule neurons transfected with U6/obsl1-1, U6/obsl1-2, or U6 control plasmid together with the GFP expression plasmid were subjected to immunocytochemistry with Fbxw8 and GFP antibodies. Representative images are shown. Scale bar = 10 µm. (TIF) [file pbio.1001060.s009.tif]

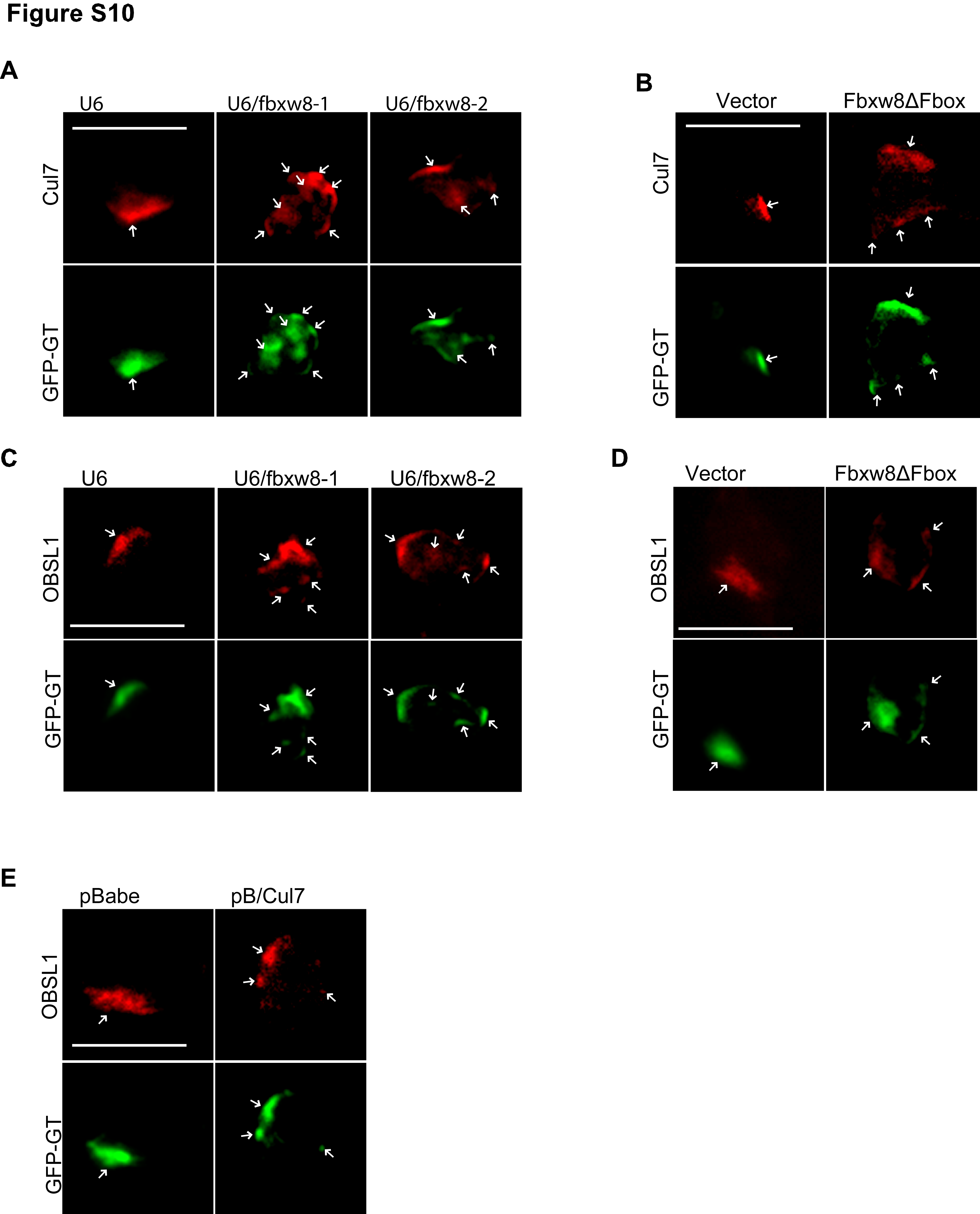

Supplement: Figure S10 — Localization of OBSL1 and Cul7 to the Golgi apparatus in neurons. (A) Granule neurons transfected with the U6/fbxw8-1, U6/fbxw8-2, or U6 control RNAi plasmid together with the Cul7-HA and GFP-GT expression plasmids were subjected to immunocytochemistry using HA and GFP antibodies. Representative images are shown. Arrows indicate co-localization of Cul7 with the Golgi apparatus. Scale bar = 10 µm. (B) Granule neurons transfected with expression plasmids encoding Fbxw8ΔFbox or control vector together with the Cul7-HA and GFP-GT expression plasmids were analyzed as in (A). Representative images are shown. Arrows indicate co-localization of Cul7 with the Golgi apparatus. Scale bar = 10 µm. (C) Granule neurons transfected with the U6/fbxw8-1, U6/fbxw8-2, or U6 control RNAi plasmid together with the V5-OBSL1 and GFP-GT expression plasmids were subjected to immunocytochemistry using V5 and GFP antibodies. Representative images are shown. Arrows indicate co-localization of OBSL1 with the Golgi apparatus. Scale bar = 10 µm. (D) Granule neurons transfected with expression plasmids encoding Fbxw8ΔFbox or control vector together with the V5-OBSL1 and GFP-GT expression plasmids were analyzed as in (C). Representative images are shown. Arrows indicate co-localization of OBSL1 with the Golgi apparatus. Scale bar = 10 µm. (E) Granule neurons transfected with the pBabe/cul7 or pBabe control RNAi plasmid together with the V5-OBSL1 and GFP-GT expression plasmids were analyzed as in (C). Representative images are shown. Arrows indicate co-localization of OBSL1 with the Golgi apparatus. Scale bar = 10 µm. (TIF) [file pbio.1001060.s010.tif]

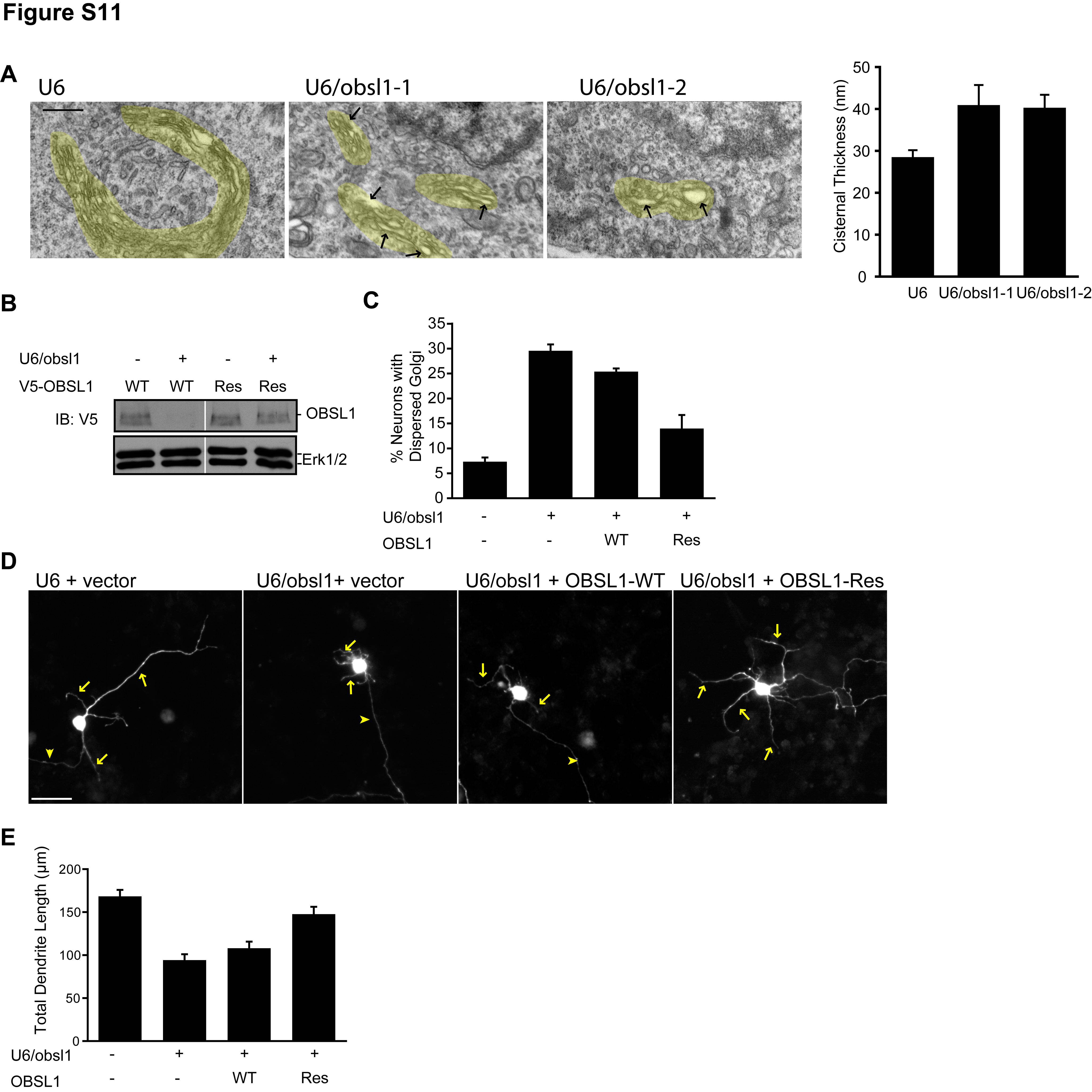

Supplement: Figure S11 — OBSL1 promotes Golgi and dendrite morphogenesis. (A) Granule neurons transfected by the nucleofection method with the U6/obsl1-1, U6/obsl1-2, or U6 control RNAi plasmid were collected after 5 d and processed for EM. In control immunocytochemical analyses, we found dispersed Golgi in 58%, 52%, and 8% of granule neurons transfected with the U6/obsl1-1, U6/obsl1-2, and control U6 plasmid, respectively. A representative EM image is shown for each condition. Scale bar = 500 nm. Right: quantification of cisterna swelling as reflected by measurement of Golgi cisternal thickness. OBSL1 knockdown significantly increased Golgi cisternal thickness (p<0.05, ANOVA followed by Bonferroni post hoc test; total cisternae measured = 219). (B) Lysates of 293T cells transfected with the V5-OBSL1-WT or V5-OBSL1-Res expression plasmids along with the OBSL1 RNAi or control U6 plasmid were immunoblotted with the indicated antibodies. (C) Granule neurons transfected at DIV2 with the U6/obsl1 or control U6 RNAi plasmid together with the expression plasmid encoding OBSL1-WT, OBSL1-Res, or control vector were analyzed as in Figure 3A. Quantification of percentage neurons with dispersed Golgi revealed that OBSL1-Res but not OBSL1-WT significantly reduced the percent of cells with dispersed Golgi in the background of OBSL1 RNAi (p<0.01, ANOVA followed by Bonferroni post hoc test; n = 3). (D) Granule neurons transfected as in (C) were analyzed as in Figure 2A. Representative images of granule neurons are shown. Scale bar = 25 µm. (E) Quantification of total dendrite length analyzed as in (D) revealed that OBSL1-Res but not OBSL1-WT significantly increased dendrite length in the background of OBSL1 RNAi (p<0.01, ANOVA followed by Bonferroni post hoc test; total neurons measured = 360). (TIF) [file pbio.1001060.s011.tif]

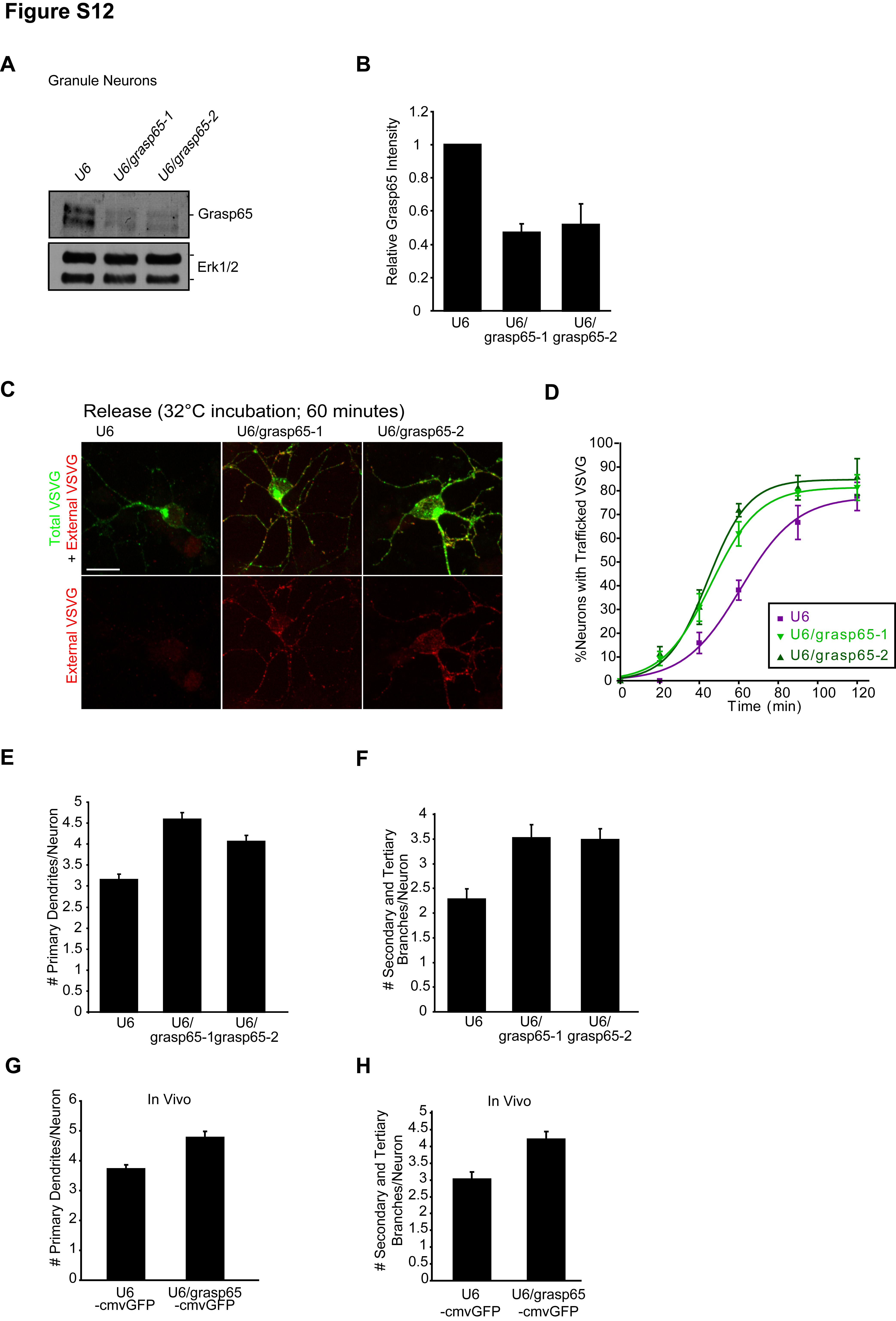

Supplement: Figure S12 — Grasp65 is a ubiquitinated target of Cul7Fbxw8 that inhibits dendrite arborization in neurons and in the cerebellum in vivo. (A) Lysates of granule neurons transfected by nucleofection method with the U6/grasp65-1, U6/grasp65-2, or control U6 RNAi plasmid were immunoblotted with the Grasp65 and Erk1/2 antibodies. Grasp65 RNAi triggered robust knockdown of endogenous Grasp65 in neurons. (B) Quantification of fold protein change of Grasp65 protein levels as compared to Erk1/2 protein levels in granule neurons transfected as in (A). Grasp65 knockdown significantly reduced Grasp65 protein levels in granule neurons (p<0.05, ANOVA followed by Bonferroni post hoc test; n = 3). (C) Granule neurons were transfected at DIV2 with the U6/grasp65-1, U6/grasp65-2, or control U6 RNAi plasmid together with an expression plasmid encoding VSVG-ts-GFP and analyzed as in Figure 3F. Representative images are shown of neurons fixed after 60 min at 32°C. Scale bar = 10 µm. (D) Quantification as in Figure 3G of neurons analyzed as in (C). Best fit curves as determined using a sigmoidal variable slope method are displayed. Grasp65 knockdown significantly increased the percentage of granule neurons with trafficked VSVG-ts at 60 min following ER release (p<0.001, two-way ANOVA followed by Bonferroni post hoc test; n = 3). (E) Quantification of dendrite branch number in granule neurons transfected with the U6/grasp65-1, U6/grasp65-2, or control U6 RNAi plasmid, analyzed as in Figure 7A, and subjected to morphometric analysis. The number of primary dendrites per neuron was significantly increased in Grasp65 knockdown neurons as compared to control U6-transfected neurons (p<0.001, ANOVA followed by Bonferroni post hoc test; total neurons measured = 294). (F) The number of secondary and tertiary dendrite branches per neuron was significantly increased in Grasp65 knockdown neurons as compared to control U6-transfected neurons (p<0.01, ANOVA followed by Bonferroni post hoc test; total neurons measu [file pbio.1001060.s012.tif]

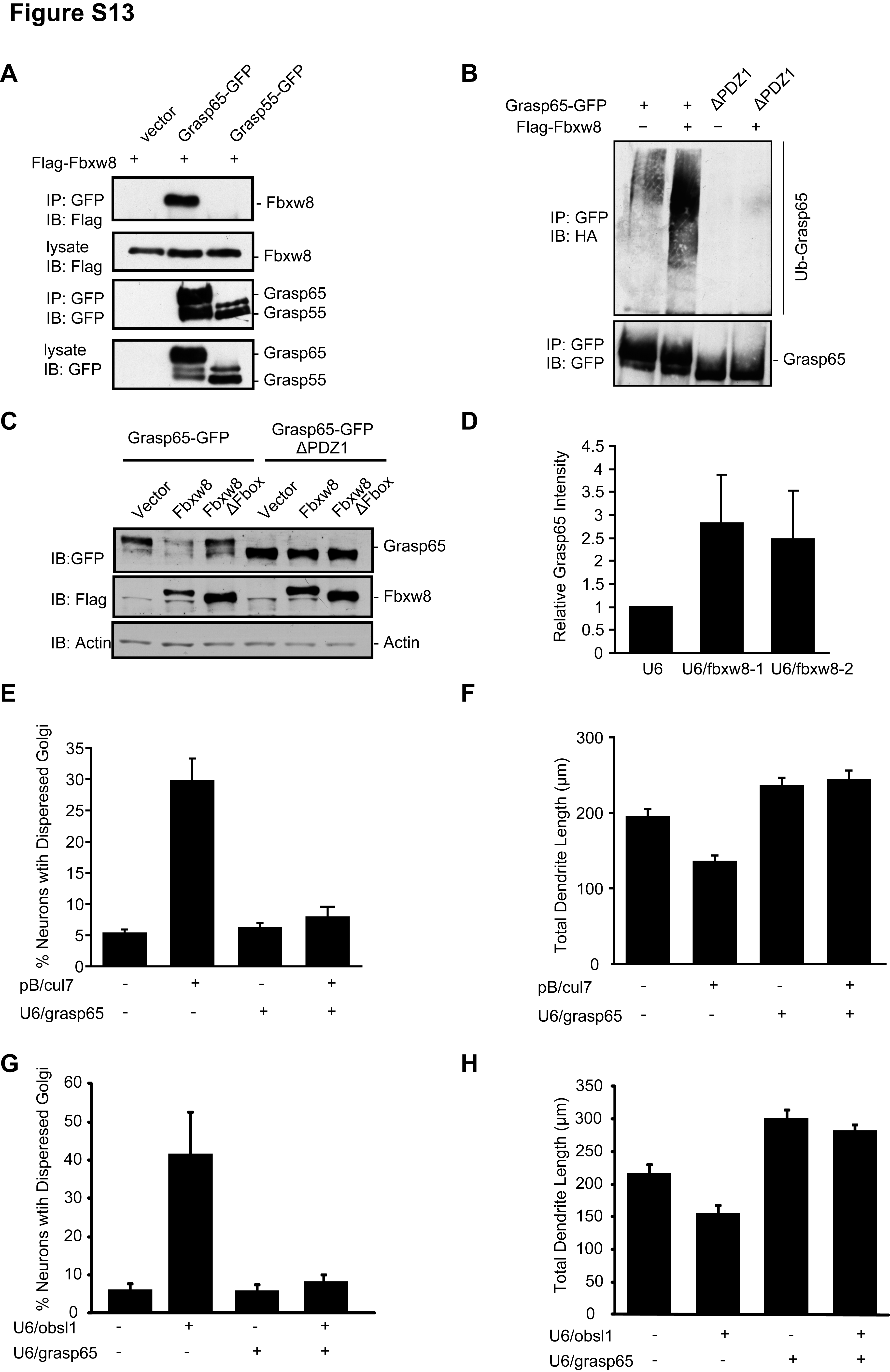

Supplement: Figure S13 — Grasp65 operates downstream of OBSL1 and Cul7Fbxw8 in the control of Golgi and dendrite morphogenesis. (A) Lysates of 293T cells transfected with the Flag-Fbxw8 expression plasmid together with the Grasp65-GFP or Grasp55-GFP expression plasmid or their control vector were immunoprecipitated with the GFP antibody. Immunoprecipitates and lysates were immunoblotted with the indicated antibodies. (B) Lysates of 293T cells transfected with the expression plasmids encoding HA-ubiquitin, Cul7, and Flag-Fbxw8 or control vector together with Grasp65-GFP or Grasp65ΔPDZ1 were incubated in 2% SDS and then diluted 1∶20 in normal lysis buffer prior to immunoprecipitation with GFP antibodies. Immunoprecipitates were immunoblotted with the HA and GFP antibodies. Fbxw8 triggered the accumulation of polyubiquitinated Grasp65 but not Grasp65ΔPDZ1 in cells. (C) Lysates of 293T cells transfected with expression plasmids encoding Grasp65-GFP or Grasp65ΔPDZ1 together with Flag-Fbxw8, a mutant lacking the F-box domain (ΔFbox), or the control vector were immunoblotted with the indicated antibodies. Fbxw8 triggered the downregulation of Grasp65 but not Grasp65ΔPDZ1 in cells. (D) Quantification of fold intensity change of Grasp65 protein levels as compared to Actin protein levels in granule neurons transfected as in Figure 7K. Fbxw8 knockdown significantly increased Grasp65 protein levels in granule neurons (p<0.05, ANOVA followed by Bonferroni post hoc test; n = 4). (E) Granule neurons transfected with the pBabe/cul7, U6/grasp65, or control pBabe or U6 plasmids, separately or in combination, were fixed and analyzed as in Figure 3A. Grasp65 knockdown significantly suppressed Cul7 knockdown-induced Golgi dispersion (p<0.001, ANOVA followed by Bonferroni post hoc test; n = 3). (F) Granule neurons transfected as in (E) and analyzed as in Figure 2A. Grasp65 knockdown significantly suppressed Cul7 knockdown-induced reduction in dendrite length (p<0.001, ANOVA followed by Bonferroni post hoc test; [file pbio.1001060.s013.tif]
